# All‐In‐One Iontronic Sensing Aligner for High‐Precision 3D Orthodontic Force Monitoring

**DOI:** 10.1002/advs.202511984

**Published:** 2025-09-08

**Authors:** Jiahao Guo, Qin Niu, Jilei Xu, Liyan Wang, Xiangyang Wang, Jiangdong Gong, Hanfei Zhu, Ruojiang Wang, Yu Chang, Tingrui Pan, Si Chen

**Affiliations:** ^1^ School of Biomedical Engineering Division of Life Science and Medicine University of Science and Technology of China Hefei 230026 China; ^2^ Center for Intelligent Medical Equipment and Devices Institute for innovative Medical Devices Suzhou Institute for Advanced Research University of Science and Technology of China Suzhou 215123 China; ^3^ Department of Orthodontics National Center for Stomatology National Clinical Research Center for Oral Diseases National Engineering Research Center of Oral Biomaterials and Digital Medical Devices Beijing Key Laboratory of Digital Stomatology NHC Key Laboratory of Digital Stomatology NMPA Key Laboratory for Dental Materials Peking University School and Hospital of Stomatology No.22, Zhongguancun South Avenue, Haidian Beijing 100081 China; ^4^ Department of Precision Machinery and Precision Instrumentation School of Engineering Science University of Science and Technology of China Hefei 230026 China; ^5^ Bionic Sensing and Intelligence Center (BSIC) Institute of Biomedical and Health Engineering Shenzhen Institute of Advanced Technology Chinese Academy of Sciences Shenzhen Guangdong 518055 China

**Keywords:** clear aligner, conformal integration, iontronic sensing, orthodontic force measurement, tooth movement

## Abstract

Clear aligners offer aesthetic and comfort advantages in orthodontics, yet their ability to deliver effective forces relies heavily on empirical judgment or large‐scale optical scanning, lacking real‐time quantitative evaluation. Integrating pressure sensors into aligners is a promising solution, but challenges in miniaturization, multi‐dimensional sensing, measurement accuracy, and biocompatibility hinder clinical application. Here, an all‐in‐one Orthodontic Force Acquisition System (OFAS) is presented that enables real‐time, 3D force monitoring using a cross‐shaped iontronic sensing array and an origami‐inspired, wireless battery‐free readout circuit miniaturized for single‐tooth placement. The system employs a cross‐linked polyelectrolyte with low creep and high repeatability, achieving 1.14% measurement accuracy. A 15‐day in vivo study demonstrates that OFAS accurately captured dynamic force profiles with strong correlation to tooth displacement (R^2^ > 0.99), confirming its feasibility for real‐time, quantitative orthodontic force measurement and enabling timely treatment adjustments.

## Graphical abstract

A wireless, battery‐free orthodontic sensing system is developed by integrating a cross‐shaped iontronic sensor and an origami‐inspired NFC circuit into clear aligners. This all‐in‐one device enables real‐time, in vivo 3D force monitoring with high precision and long‐term stability, providing clinicians with quantitative biomechanical feedback to optimize treatment efficacy and personalize orthodontic care.

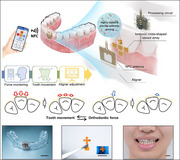

## 1 Introduction

**Figure 1:**
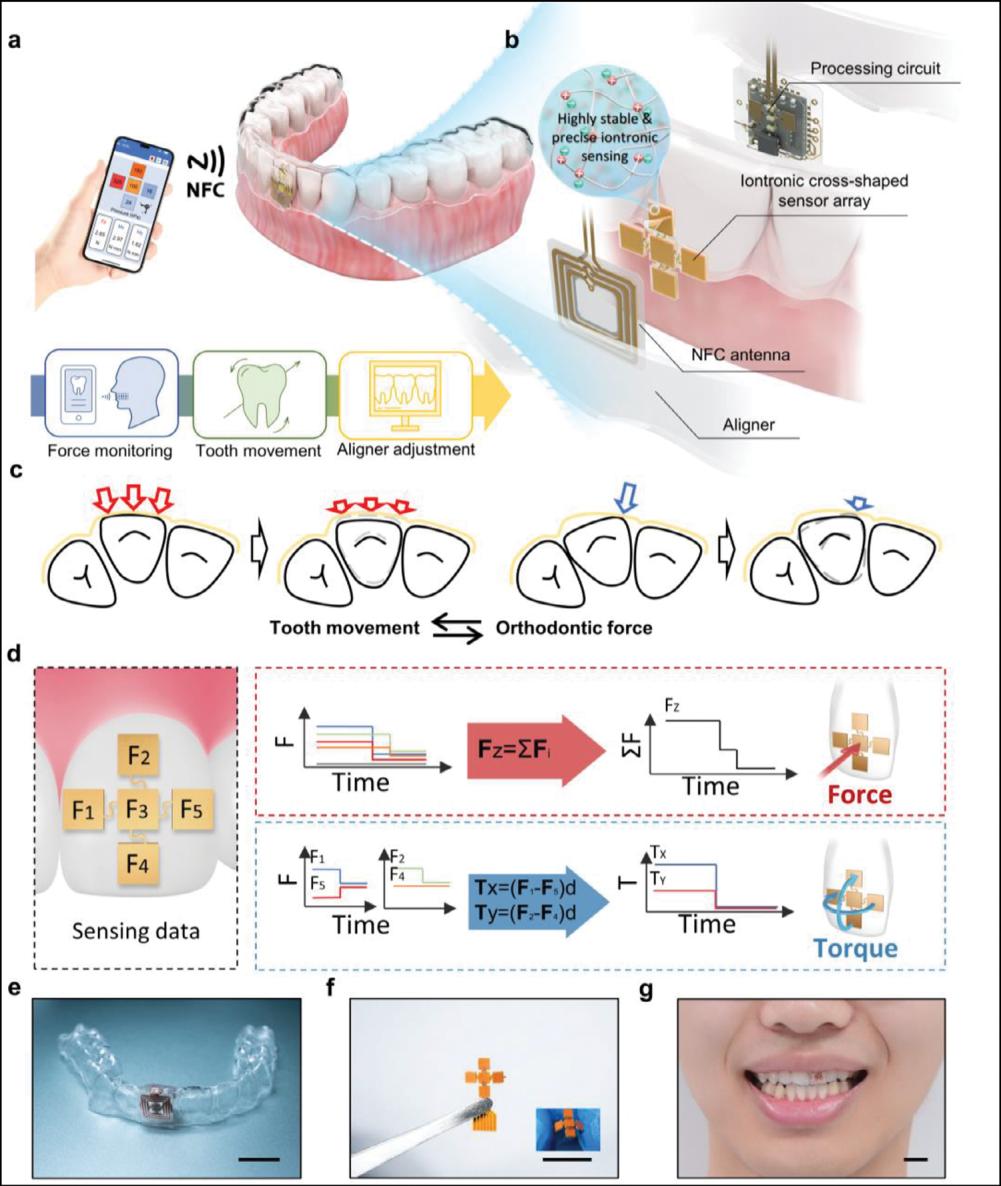
a) Schematic illustration of the system. 3D orthodontic force signals acquired by a passive cross‐shaped sensing array are wirelessly transmitted to the host system via NFC, enabling real‐time assessment of tooth movement and force distribution, which can guide clinicians in aligner adjustment. b) Exploded view of the smart orthodontic aligner, illustrating the integration of sensing and circuitry modules encapsulated between inner and outer layers of the aligner that retain orthodontic functionality. c) Diagram of the relationship between orthodontic force and tooth movement working principle of force and torque sensing. d) By capturing spatially distributed pressure data from the sensing array, the system simultaneously extracts 3D orthodontic force information. e) Photograph of the OFAS device prototype. Scale bar, 1cm. f) Photograph of the cross‐shaped iontronic sensing array. The inset displays a photo of the sensor in a bent state. Scale bar, 1 cm. g) Photograph of an OFAS worn by a participant. Scale bar, 1 cm.

Malocclusion is one of the most prevalent oral health conditions globally, with epidemiological studies reporting a prevalence of up to 56% among children and adolescents.^[^ ^1^ ^]^ Malocclusion significantly compromises oral health, potentially leading to adverse outcomes such as masticatory difficulties, speech impairments, periodontal disease, and temporomandibular joint disorders.^[^ ^2^, ^3^ ^]^ In recent years, clear aligners, represented by Invisalign, have been widely adopted in clinical practice as an alternative to traditional fixed appliances, owing to their aesthetic appeal, comfort, and ease of maintenance. These advantages have led to widespread acceptance among both patients and clinicians.^[^ ^4^, ^5^ ^]^ Despite their popularity, compared to the mature fixed orthodontic system, clear aligners have narrower indications, longer treatment durations, and lower clinical efficacy in complicated cases. The pivotal determinant lies in the lack of clear mechanical properties and quantitative assessment of the corresponding tooth movement. From a mechanical perspective, a dynamic feedback loop exists between orthodontic force and tooth position: while forces induce tooth movement, changes in tooth position subsequently alter the mechanical interaction between the aligner and dentition, modifying the applied force vectors (**Figure** **1**c). Whether the teeth are moving as intended is a key criterion for evaluating the effectiveness and appropriateness of the current stage of orthodontic treatment.^[^ ^5^, ^6^, ^7^ ^]^ Therefore, real‐time monitoring not only evaluates treatment efficiency but also provides clinical guidance for appliance design and replacement, thereby enhancing treatment efficacy, shortening treatment duration, and expanding the applicability of clear aligner therapy as well as minimizing adverse effects such as pain or root resorption.^[^ ^1^, ^8^, ^9^ ^]^ Despite technological advances, force management in clear aligner therapy remains predominantly experience‐based, highlighting a critical unmet need for reliable in vivo real‐time 3D force monitoring systems.^[^ ^10^, ^11^ ^]^

To meet the demand for real‐time monitoring of orthodontic forces in clear aligner therapy, four core technical challenges must be addressed: multi‐dimensional force sensing, wearable adaptability, measurement precision, long‐term stability, and biocompatibility.^[^ ^12^, ^13^ ^]^ This represents not only a critical technical barrier to achieving precise biomechanical feedback in orthodontics, but also a core challenge limiting the advancement of clear aligner technology toward true in vivo measurement. At first, precise treatment necessitates sensors capable of detecting not only unidirectional forces but also torsional and tilting moments, as teeth exhibit complex 3D movement patterns under aligner pressure, leading to the conclusion that the detection of multi‐dimensional forces is critical. Additionally, the sensing system must be seamlessly integrated into aligners without compromising their thin and flexible structure or patient comfort. Moreover, given the subtle nature of orthodontic forces, sensors must demonstrate high repeatability and minimal signal drift to ensure precise detection of orthodontic forces throughout the entire treatment period. Finally, the system must endure prolonged exposure to saliva, temperature fluctuations, and mechanical stress while remaining non‐irritating to oral tissues during months of continuous use.

Although several in vivo, real‐time orthodontic force monitoring approaches for clear aligners have been proposed in recent years, significant limitations remain. Hao Feng employed a mechanoluminescent approach to measure orthodontic forces; however, due to the short duration of luminescence and high susceptibility to external light interference, accurate measurements are difficult to achieve.^[^ ^14^ ^]^ Zamani inserted Prescale Film between the clear aligner and a dental model to estimate orthodontic force based on the color intensity changes of the film. While this method provides a visual representation of approximate force distribution, it is limited to single‐use measurements, and the film's thickness interferes with the basic orthodontic function of the aligner.^[^ ^15^ ^]^ In addition, spatial constraints of electronic circuits and the reliance on batteries severely limit the practical application of real‐time monitoring systems in intraoral environments. For example, Hao Feng's BLE‐based system required battery power and occupied substantial intraoral space, compromising both wearing comfort and clinical feasibility.^[^ ^16^ ^]^ Although wireless passive solutions that integrate LC resonant circuits with flexible pressure sensors have facilitated device miniaturization and enabled passive operation, the inherent limitations of circuit design make it challenging to simultaneously drive multiple sensors within a confined space. This limitation complicates the performance of 3D force measurements on target teeth. Furthermore, these systems demand a precise controlled measurement environment, thereby restricting their practical applicability.^[^ ^11^, ^17^ ^]^ Overall, existing technologies fail to simultaneously meet the comprehensive requirements of 3D real‐time monitoring, miniaturization, measurement precision, and long‐term stability in clear aligner treatment.

To overcome these limitations, we introduce the Orthodontic Force Acquisition System (OFAS)—a wireless, battery‐free, and all‐in‐one platform for in vivo 3D orthodontic force monitoring embedded within clear aligners (Figure 1a). The system employs a cross‐shaped sensing array design with five independent iontronic sensing units to enable 3D monitoring, capturing both force and torque information within the target region (Figure 1d). A fully cross‐linked polyelectrolyte elastomer is utilized in the sensing unit to establish an iontronic interface with the electrodes. This design ensures creep‐free and highly repeatable behavior, enabling the sensor to consistently provide stable and accurate electrical signals over extended periods. Additionally, the system incorporates a flexible signal acquisition and analog‐to‐digital conversion circuit, integrated with an NFC‐based wireless passive power supply and signal transmission mechanism. To reduce the circuit size, an origami‐inspired design is applied to the flexible circuit, resulting in a total circuit area of less than 8 × 8 mm^2^, which facilitates seamless integration onto a single tooth. Furthermore, the entire system is integrated into the aligner via conformal thermoplastic encapsulation between dual‐layer aligners, ensuring precise adhesion to the tooth surface while retaining the essential orthodontic functionality of the aligner. Additionally, this packaging approach enables the system to achieve long‐term stability within the complex oral environment by hermetically sealing the sensor and circuit within a biocompatible material. A 15‐day in vivo experiment was performed to validate the efficacy of OFAS for achieving real‐time 3D monitoring of orthodontic forces. The measured force data exhibited strong correlation with spatial tooth movement information obtained through intraoral scanning (R^2^ > 0.99), thereby clarifying the effectiveness and appropriateness of each stage of tooth movement. This provides a reliable foundation for clinicians to make precise adjustments to treatment plans. Compared with other orthodontic measurement devices based on clear aligners (Table S1, Supporting Information), this work addresses key challenges including wearable adaptability, 3D force sensing, measurement precision, long‐term stability, and biocompatibility.

## 2 Results and Discussion

### 2.1 Design of the OFAS

The OFAS is a highly integrated wireless passive 3D orthodontic force sensing system. OFAS consists of a sensing unit, an antenna, and a processing circuit. The sensing unit primarily consists of a drift‐free, wide‐range cross‐shaped iontronic sensing array, which is interconnected through serpentine wiring. The mechanical properties of the sensing material are optimized via crosslinker modulation to meet the requirements of multi‐dimensional, high‐magnitude force measurements on teeth. The system also incorporates an NFC‐based passive signal acquisition and transmission circuit, as well as a dual‐layer thermoplastic film that retains orthodontic functionality (Figure 1b). The cross‐shaped sensing array comprises five sensing points (Figure 1f), enabling calculation of the vertical force component (F_z_) through summation. The torque components (M_x_ and M_y_) are derived by computing the force differences between left/right and top/bottom sensor points, respectively, and multiplying by their distances from the central point (Figure 1d).

The entire circuit is highly integrated and can be encapsulated within the space of a single incisor in the invisible aligner to ensure wearing comfort (Figure 1e). These data can be transmitted back to the orthodontist, enabling a more precise understanding of the orthodontic forces and tooth movement, thereby facilitating the development of optimized treatment plans.

To the best of our knowledge, the OFAS system developed in this work represents the first fully wearable, wireless, and passive orthodontic sensing platform that simultaneously achieves a wide measurement range and high precision. In comparison to existing technologies, it exhibits clear advantages across multiple dimensions, including sensing capability, wearability, measurement range, accuracy, and other critical performance metrics (Table S1, Supporting Information).

### 2.2 Cross‐Shaped Iontronic Sensing Array for High‐Precision Force Detection

Orthodontic force measurement in clear aligners presents multiple challenges. From a biomechanical perspective, the pressure exerted by clear aligners exhibits a markedly non‐uniform distribution, with localized pressures potentially reaching the MPa range. This necessitates that the sensing system possess a wide detection range to accurately capture the highly variable mechanical signals involved. Considering that a single aligner is typically worn for 1–2 weeks during clinical treatment, and must provide continuous mechanical stimulation to the teeth, the sensing system is also required to demonstrate long‐term stability for continuous monitoring.^[^ ^18^ ^]^ In addition, the mechanical stress induced by repeated daily removal and reinsertion of the aligner imposes stringent demands on the cyclic durability of the sensing unit.^[^ ^13^ ^]^ Notably, the curved morphology of the tooth crown introduces deformation at the contact interface, posing further challenges to the interfacial adaptability of the sensor electrodes.

To address the specific requirements of orthodontic force monitoring in clear aligner therapy, this study proposes a cross‐shaped iontronic sensing array (CISA) featuring a sandwich‐structured design interconnected via serpentine traces. The array consists of copper‐gold electrodes on a polyimide (PI) substrate and a microstructured polyelectrolyte elastomer (PEE) membrane (Figure 3a, Figure S1, Supporting Information). Iontronic sensing is an ideal choice for this application, as its electric double‐layer capacitance mechanism enables a more sensitive response to static pressure per unit area compared to conventional capacitive sensors.^[^ ^19^, ^20^, ^21^ ^]^ In addition, microstructures on the elastomeric membrane enhance sensitivity in the low‐pressure regime, while the material design prevents signal saturation under high‐pressure conditions, thereby ensuring consistent and reliable performance across a broad pressure range.^[^ ^22^ ^]^

**Figure 2:**
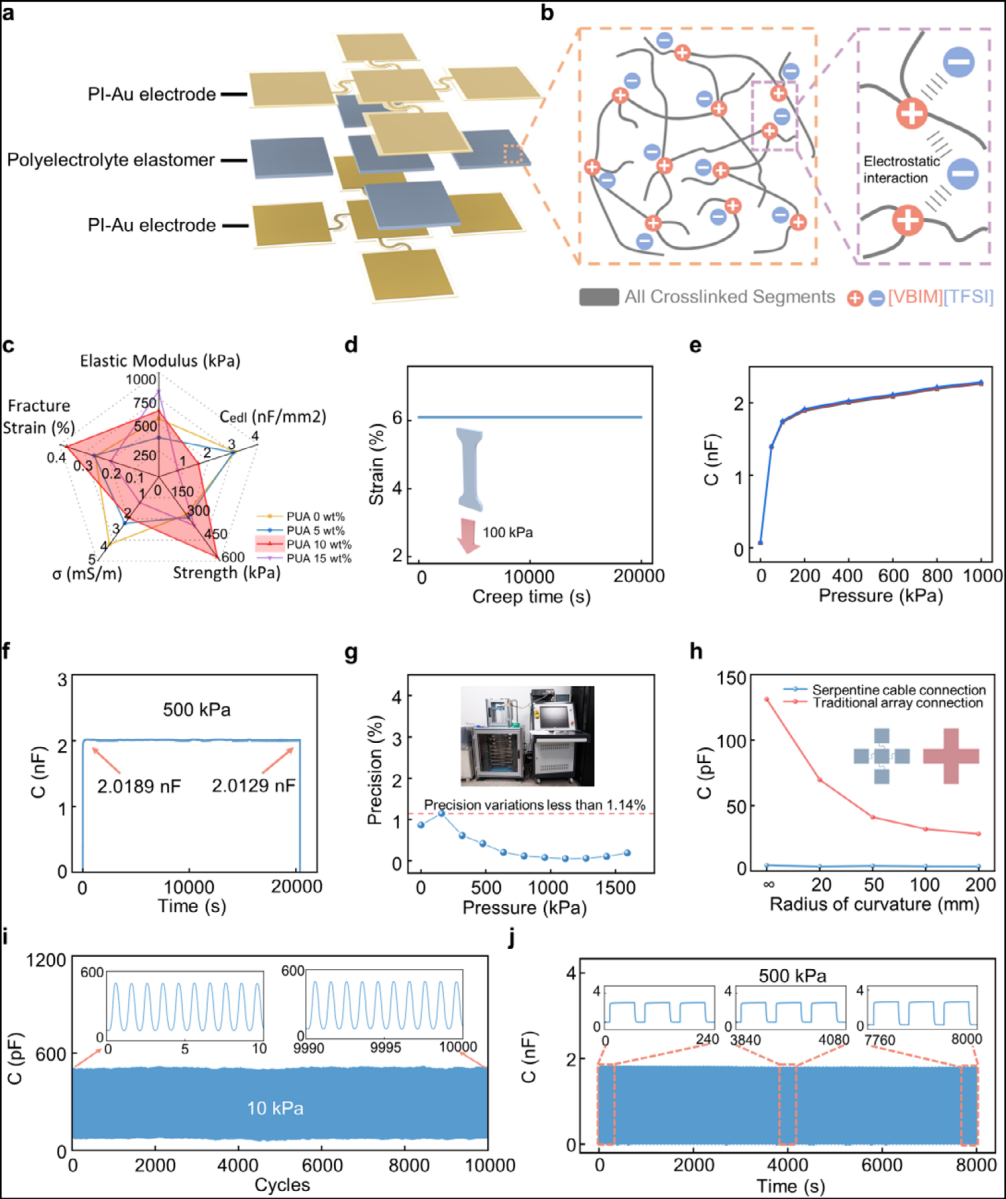
a) Schematic illustration of the composition and working principle of the PEE‐based CISA. b) Chemical structure of the PEE and its mechanical functionalities. c) Radar plot depicting the 5D performance metrics of PEE samples with varying crosslinking densities. d) Creep strain of the PEE over time under a constant stress of 100 kPa. e) Capacitance–pressure response curve of the CISA (*n* = 3). f) Time‐resolved stability of the CISA under static compression at 500 kPa for 20 000 s. g) The accuracy of the sensor under different pressures. h) Initial capacitance variations of two CISA configurations adhered to surfaces with different curvature radii (red: conventional integrated circuit layout; blue: serpentine interconnect design). i) Output stability of the CISA over 10 000 cycles under sinusoidal mechanical loading at 10 kPa and 1 Hz. j) Output stability of the CISA over 100 cycles of square‐wave loading at 500 kPa.

The PEE serves as the core component of the OFAS sensing unit, critically determining its sensing performance. To ensure stability and biocompatibility, all constituents of the PEE were designed as crosslinkable materials free of free‐dispersed species (**Figure** **2**b). In addition, electrostatic interactions were employed to immobilize anionic groups within the copolymer network onto the polymer backbone, effectively preventing ion leakage. Moreover, the incorporation of 2‐methoxyethyl acrylate (MEA) as a neutral segment mitigates interchain interactions, reduces viscoelasticity, and suppresses creep behavior, thereby enhancing the long‐term reliability of measurements.^[^ ^21^ ^]^

Given the substantial stresses that may arise during the thermoforming process and daily wear of clear aligners, the membrane material must exhibit high mechanical robustness to prevent fracture or damage. The proportion of polyurethane acrylate (PUA), owing to its long‐chain structure, plays a critical role in determining the suitability of the PEE for orthodontic force sensing in clear aligner applications. By tuning the mass ratio of PUA within the composite, the mechanical properties—including tensile strength, elastic modulus, and fracture strain—were systematically optimized. Comparative analysis of materials with varying PUA content revealed that a PUA mass ratio of 10 wt.% achieves the optimal balance between mechanical and electrical performance of the sensor (Figure 2c; Figures S2–S6, Supporting Information). Under 100 kPa continuous loading for over one week in ambient conditions with fluctuating temperature and humidity, the PEE material exhibited excellent environmental stability, with no detectable mass change, leakage, or moisture uptake (Figure S7, Supporting Information). In addition, the creep behavior of stretched samples under a constant tensile load of 100 kPa was evaluated, revealing that the strain stabilized at ≈6.1% over a duration of 20 000 s (Figure 2d).

Following the optimization of the PEE composition and its corresponding functional properties, the sensor underwent three cycles of pressure–capacitance characterization. As shown in Figure 2e, the device exhibited stable and reliable performance under pressures up to 1 MPa. To assess its suitability for long‐term, high‐precision orthodontic monitoring, the sensor's output stability was further evaluated under a sustained pressure of 500 kPa. As depicted in Figure 2f, during a prolonged loading test lasting over 20 000 s, the signal variation remained within 0.3%, demonstrating excellent operational stability for extended force monitoring. Moreover, precision characterization using a high‐accuracy dead‐weight force standard revealed a maximum measurement deviation of less than 1.14%, indicating outstanding repeatability (Figure 2g). Additionally, the response and recovery times were both below 50 ms, satisfying the real‐time performance requirements for dynamic orthodontic force monitoring (Figure S8, Supporting Information).

In the design of the cross‐array sensor, serpentine interconnects were employed to connect individual sensing units, enabling improved conformability to curved surfaces and minimizing the impact of surface curvature on sensor performance.^[^ ^23^, ^24^, ^25^ ^]^ Across tests conducted on surfaces with five different curvature diameters, conventional electrode designs exhibited significant capacitance changes under bending, with an initial capacitance variation reaching up to 103 pF on a surface with a 10 mm curvature radius. In contrast, the sensor with serpentine interconnects showed negligible sensitivity to curvature‐induced deformation, with initial capacitance variations of less than 1 pF (Figure 2h), blue: serpentine design; red: conventional electrode structure).

Additionally, to evaluate the potential impact of repeated aligner insertion and removal during prolonged use, cyclic loading tests were conducted under both low and high pressure conditions. The low‐pressure test involved a 1‐s cycle at 10 kPa (Figure 2i), while the high‐pressure test simulated wear conditions with a 90‐s cycle comprising 60 s of loading at 500 kPa followed by 30 s of unloading (Figure 2j). The results demonstrate that the sensor maintains excellent stability under both short‐cycle low‐pressure and long‐cycle high‐pressure conditions.

### 2.3 Characterizations of the Wireless, Battery‐Free Orthodontic Monitoring System

Conventional orthodontic testing devices are typically bulky and prone to causing soft tissue irritation, which significantly undermines patient compliance during long‐term use. This technical bottleneck directly hinders both the clinical application and experimental progress of precision orthodontic treatments. Additionally, some studies have attempted to insert sensing units between clear aligners and the tooth surface. While structurally simple, this approach introduces a non‐negligible thickness that not only compromises the intended biomechanical function of the aligners but also results in force measurements that poorly reflect the actual orthodontic forces exerted on the teeth.^[^ ^24^ ^]^

#### 2.3.1 Origami‐Inspired Design for Miniaturing OFAS

**Figure 3:**
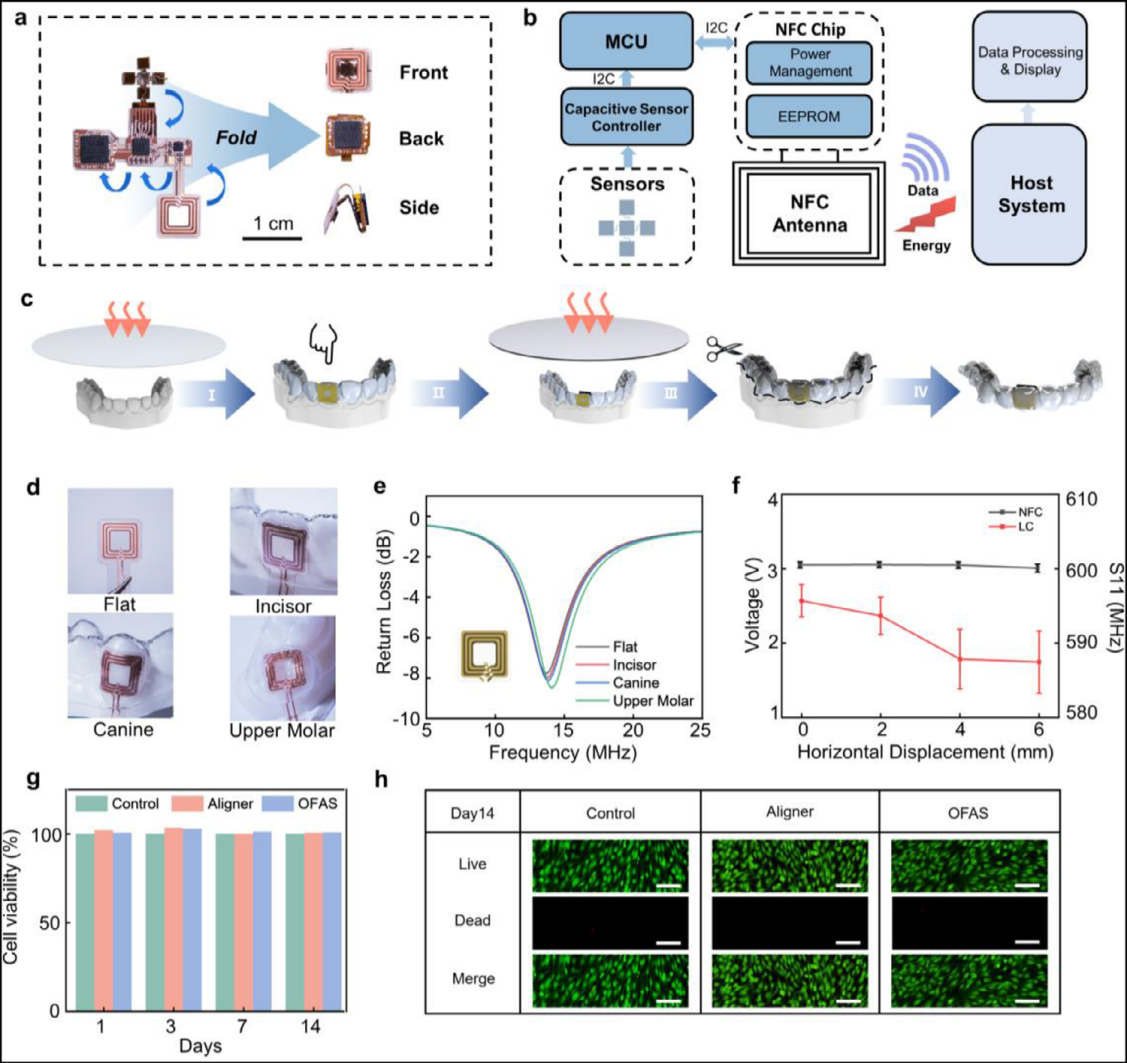
a) Schematic illustration of the folding structure of the flexible circuit. b) Functional block diagram of the complete orthodontic measurement platform. The OFA system comprises a sensing array, capacitive signal acquisition module, control unit, and NFC antenna, with data wirelessly transmitted to the host system via NFC. c) Schematic diagram of the system packaging process. d) Schematic showing antenna placement on various tooth surfaces. e) Variation in reflection loss (RL) of the antenna at different tooth positions. f) Comparison of measurement stability between NFC‐ and LC‐based signal transmission schemes under varying spatial misalignments, using antennas of the identical size. Points, mean; error bars, s.d.; *n* = 3. g) Cell viability evaluation of OFAS and orthodontic aligners without integrated circuit. h) AO/EB staining assay of HGF cells cultured with different sample types. Scale bar, 100 µm.

To address the clinical demand for miniaturized devices in orthodontic treatment, this study adopts an origami‐inspired design to achieve circuit compression. While maintaining the full functionality of an NFC‐based wireless, battery‐free, multi‐channel signal acquisition system, the circuit footprint is reduced from 35 mm × 25 mm to just 8 mm × 8 mm. This significant miniaturization not only enhances wearing comfort and aesthetics but also enables the integration of the entire system onto the surface of a single tooth crown (**Figure** **3**a). The OFAS comprises a multi‐channel signal acquisition module, a microcontroller unit, an NFC chip, sensing units, and a transmission coil. The corresponding host system utilizes a magnetic inductive coupling mechanism to achieve both wireless power transfer and data acquisition, enabling real‐time analysis of the current orthodontic force and tooth movement status. This design eliminates the need for onboard batteries, thereby conserving space and avoiding potential safety concerns associated with embedded power sources.^[^ ^26^ ^]^ The acquired data can be fed back to clinicians to provide a more precise assessment of the orthodontic status, enabling more informed adjustments to treatment plans (Figure 3b; Figure S9, Supporting Information).

#### 2.3.2 All‐In‐One Integration of OFAS with Clear Aligner

To seamlessly integrate the orthodontic function of the aligner with force measurement capabilities during treatment, we implemented the OFAS integration strategy, as illustrated in Figure 3c. The sensing circuit is encapsulated between two layers of thermoplastic material via a dual‐layer thermoplastic conformal encapsulation strategy, allowing the measurement system to conform intimately to the tooth surface. This approach not only enables real‐time monitoring of forces exerted on the teeth but also ensures patient comfort and therapeutic efficacy. This design preserves the aligner's inherent orthodontic functionality while enabling accurate and reliable force quantification throughout the treatment process.

#### 2.3.3 Robust NFC‐Based Wireless Transmission in Diverse Oral Environments

To meet the engineering requirements of an oral biomechanical monitoring system, the robustness of the wireless transmission link is a critical factor affecting overall system reliability. To ensure effective signal transmission regardless of the orthodontic state or anatomical variability of different teeth (incisors, canines, and molars), the antenna must maintain functionality despite deformation. To evaluate this, we placed the antenna coil on various representative surfaces—including a flat plane, the labial surface of an incisor, the surface of a canine, and the occlusal surface of a molar—and measured its response using a vector network analyzer (VNA), as shown in Figure 3d. The reflection loss (RL) results in Figure 3e indicate that the resonance frequency shifts minimally across these placements, satisfying the antenna positioning requirements for various orthodontic scenarios.

To further assess the transmission stability of the NFC‐based system, we compared its sensing performance against that of an LC (inductor‐capacitor) circuit‐based configuration. LC single‐point sensors were fabricated using the same antenna dimensions and sensing area, as well as the identical PEE‐sensitive layer (Figure S10, Supporting Information). Experimental results revealed that the LC‐based device exhibited significant frequency drift under spatial misalignment between the transmitting and receiving antennas. In contrast, the NFC‐based system maintained superior transmission stability and signal integrity under the same conditions, as illustrated in Figure 3f, making it more suitable for reliable and accurate data transmission in dynamic oral environments.

#### 2.3.4 Biocompatibility of OFAS

To ensure the biocompatibility of the OFAS, we selected human gingival fibroblasts (HGFs), the primary cell type in oral soft tissue, commonly used to evaluate the biocompatibility of dental materials due to their direct clinical contact with gingiva and potential exposure to materials released from orthodontic appliances.^[^ ^27^ ^]^ HGF cell suspensions were seeded onto membranes with and without the ionic coating. Cell viability was assessed using the Cell Counting Kit‐8 on days 1, 3, 5, and 7 post‐culture. As shown in Figure S11 (Supporting Information), HGFs exhibited similar growth trends on both coated and uncoated membranes, with no statistically significant differences compared to the control group. Additionally, system‐level biocompatibility was evaluated via cell viability assays and fluorescence staining, as shown in Figure 3g,h. The results showed that HGF growth on the OFAS, the aligner, and the blank control samples was comparable, with no significant differences observed. These results confirm that the system exhibits excellent biocompatibility, supporting its safe use during long‐term wear.

### 2.4 Orthodontic Process Monitoring Using OFAS on a Dental Simulator

**Figure 4:**
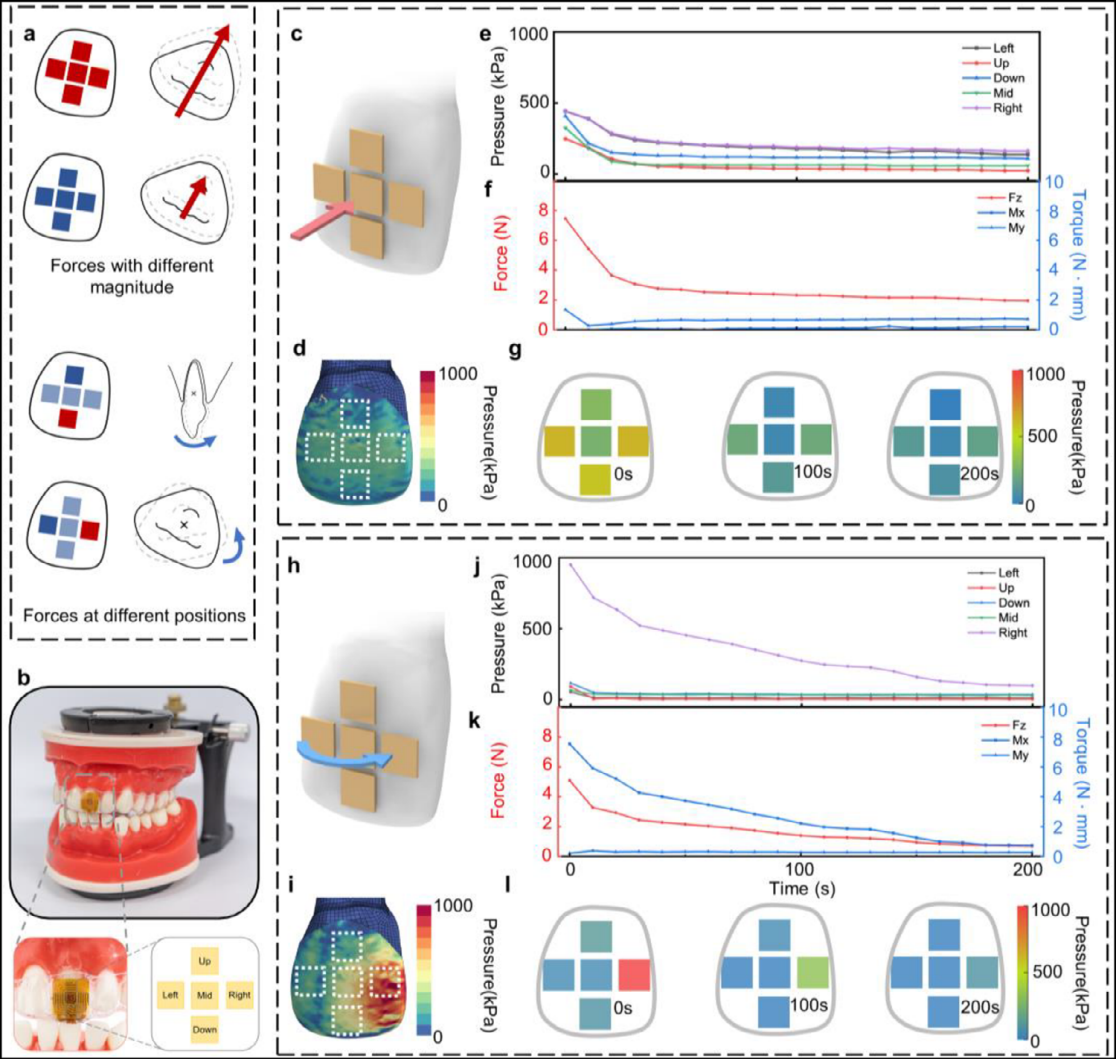
a) Schematic illustration of the force applied to a tooth, demonstrating the OFAS's capability to determine both the magnitude and direction of the applied force. b) Photograph of the typodont‐based experimental setup, showing the spatial distribution and labeling of the five sensing points. c) Schematic of the typodont model designed to simulate translational tooth movement. d) Finite element simulation of the initial stress distribution. e) Pressure response data of CISA. f) Corresponding time‐dependent force and torque curves. g) Orthodontic force heatmaps at 0, 100, and 200 s. h) Schematic of the typodont model designed to simulate rotational tooth movement. i) Finite element simulation of the initial stress distribution. j) Pressure response data of CISA. k) Corresponding time‐dependent force and torque curves. l) Orthodontic force heatmaps at 0, 100, and 200 s.

In orthodontic treatment, precise monitoring of mechanical changes during tooth movement is critical for both therapeutic efficacy and patient experience. Excessive initial force may cause significant discomfort or even adverse outcomes such as root resorption, whereas insufficient force may fail to induce effective tooth displacement.^[^ ^28^ ^]^ The CISA enables the measurement of both force and torque applied to the teeth, thereby facilitating the assessment of tooth movement trajectories and their alignment with the planned treatment path (**Figure** **4**a). Moreover, when the measured force diminishes to a low and stagnant level, it indicates that the aligner is no longer exerting effective orthodontic force, signaling the need for timely replacement. Real‐time monitoring of force states not only aids in optimizing treatment protocols but also provides orthodontists with accurate, data‐driven insights, ultimately reducing the risk of complications and enhancing treatment efficiency.

Figure 4b illustrates the orthodontic monitoring simulation system based on a typodont model, along with the distribution and labeling of the five sensing points in the CISA. The typodont—a commonly used dental training device—serves as a surrogate for simulating tooth movement. By replacing the dental arch with a wax‐based model, the OFAS can be mounted onto the typodont to enable rapid, short‐term simulation and validation of orthodontic force dynamics and tooth movement patterns.^[^ ^29^ ^]^

In orthodontic treatment, tooth movement primarily occurs as a combination of two fundamental modes: rotation and translation. This study focuses on analyzing two common types of tooth movement during orthodontic procedures: rotational movement around the tooth root and translational movement of the entire crown in the lingual direction.

As shown in Figure 4c–l, the pressure variations across different channels, the corresponding force and torque evolution, the initial force distribution simulation, and pressure heatmaps at 0, 100, and 200 s for each sensing point are presented for both translational and rotational tooth movements. During translational movement, a significant change is observed only in F_z_, corresponding to the bodily inward movement of the tooth crown. These findings demonstrate that the OFAS (Oral Functional Aligner System) is capable of effectively distinguishing and analyzing different modes of tooth movement. In contrast, during rotational motion, the force–torque curves indicate a gradual decrease in M_x_ and F_z_ over time, while M_y_ remains nearly constant, suggesting that the tooth primarily undergoes rotation along the root axis—consistent with the experimental design.

### 2.5 In Vivo Experiment Using OFAS for Orthodontic Process Monitoring

**Figure 5:**
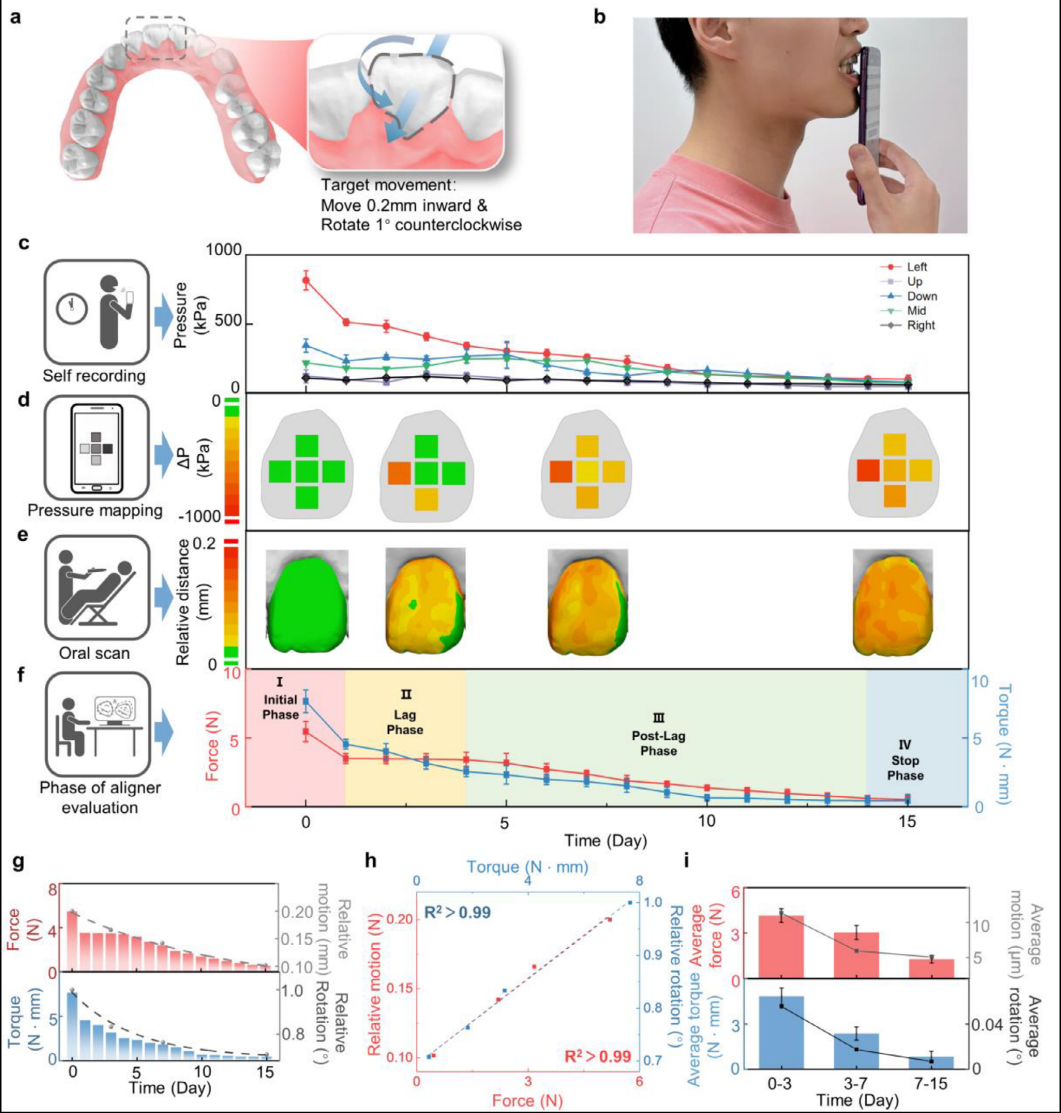
a) Schematic illustration of the target tooth displacement. b) Diagram of the 15‐day in vivo testing protocol. c) Time‐resolved pressure data from each sensing channel over the 15‐day observation period. Points, mean; error bars, s.d.; *n* = 3. d) Force distribution heatmaps corresponding to days 0, 3, 7, and 15; e) Intraoral scan models on days 0, 3, 7, and 15, illustrating the staged tooth movement; f) Force and torque data computed by the sensing system, corresponding to different orthodontic stages. Points, mean; error bars, s.d.; *n* = 3. g) Changes in overall force and torque applied to the tooth over 15 days, and the corresponding translational and rotational displacement of the tooth relative to the target position on days 0, 3, 7, and 14. Dashed lines represent curve fits for translation and rotation trajectories. h) Correlation analysis between orthodontic force/torque and the resulting translational/rotational displacement on days 0, 3, 7, and 15. i) Average force and torque, along with corresponding displacement and rotation angle change rates, measured over three intervals: days 0–3, 3–7, and 7–15. Bar height, mean; error bars, s.d.; *n* = 3.

To further evaluate the clinical utility of OFAS in monitoring orthodontic forces exerted by clear aligners and the accuracy of the system's measurements, a 15‐day in vivo orthodontic monitoring study was conducted with a volunteer. Based on the subject's dentoalveolar condition, the treatment goal was to achieve a 0.2 mm lingual translation and a 1° counterclockwise rotation of the upper left central incisor (tooth #21), as illustrated in **Figure** **5**a. The customized OFAS was fitted intraorally (Figure 5b). The experimental protocol required the subject to perform multiple measurements and record data daily between 12:00 and 14:00. To verify whether OFAS accurately captured the trend of tooth displacement during orthodontic treatment, intraoral 3D scans were performed on days 0, 3, 7, and 15 to track changes in the relative position of the target tooth.

#### 2.5.1 In‐Vivo Data Acquisition by OFAS

Figure 5c depicts the pressure variation curves recorded by each OFAS sensing channel over a continuous 15‐day monitoring period. As shown, the Left channel consistently exhibits significantly higher pressure levels compared to other channels, with a marked decline over time. Between days 2 and 5, an increase in pressure is observed across the remaining channels, which may be attributed to rapid tooth rotation leading to elevated stress on adjacent sensing regions.

Figure 5d presents heatmaps of the pressure distribution across sensing points on days 0, 3, 7, and 15, offering an intuitive visualization of the spatial evolution of pressure during treatment. In parallel, Figure 5e shows oral scan models captured on days 0, 3, 7, and 15. Comparative analysis with the baseline model from day 0 reveals a clear trend of tooth displacement. Notably, the application of greater force on the left side induced a pronounced rotation of the corresponding tooth, accompanied by an overall posterior translational movement.

#### 2.5.2 The Classification of Orthodontic Treatment Stages Based on OFAS Data Analysis

Figure 5f illustrates the temporal evolution of overall force of bodily and rotational movement, calculated with respect to the central point of the OFAS sensing surface as the origin. Analysis of the total force reveals a distinct pattern: a sharp decrease from day 0 to day 1, followed by a relatively stable force level between days 1 and 4. After day 4, the force exhibits a continuous and significant decline, ultimately dropping below 0.2 N by day 14—corresponding to a pressure lower than that of capillary blood pressure (20–26 g cm^−^^2^). At this stage, the force applied to the tooth is considered insufficient to induce further meaningful displacement. These changes in force magnitude correspond closely to the distinct biological stages of tooth movement: **Phase I (initial phase)** – characterized by rapid physical displacement of the tooth within the periodontal space; **Phase II (lag phase)** – marked by hyalinization of the periodontal ligament and undermining resorption of alveolar bone, during which tooth movement is minimal; **Phase III (post‐lag phase)** – as bone resorption progresses, tooth movement gradually resumes; **Phase IV (plateau phase)** – where OFAS pressure data stabilizes at a low level, indicating a diminished mechanical effect of the aligner on the tooth. This plateau suggests that the therapeutic force has substantially weakened, signaling the appropriate time to replace the aligner for continued treatment efficacy.^[^ ^30^, ^31^ ^]^

Interestingly, between days 2 and 4, distinct pressure variations were observed across the sensing channels: the pressure in the left channel decreased, while pressures in the other channels increased, indicating a prominent rotational movement. However, as shown in Figure 5d, the average pressure during this period remained nearly unchanged. Through innovative 3D force mapping, we mechanistically decoded rotational relapse by capturing the dynamic interplay between applied de‐rotational forces and the PDL's restorative stress generation. This antagonistic biomechanical relationship, quantitatively established in our study, explains the well‐documented instability of derotated teeth in clinical practice.

#### 2.5.3 Correlation Analysis of Tooth Forces and Displacement Changes

Figure 5g illustrates a clearer correlation between force/torque and translational/rotational tooth movement. The bar graphs represent changes in force and torque over a 15‐day period, while the four discrete points indicate displacement and rotation angle of the target tooth's centroid at days 0, 3, 7, and 15, as determined by intraoral scanning. The dashed line denotes a fitted curve based on these four data points. The observed trends demonstrate a strong concordance between variations in mechanical loading and corresponding positional and angular changes, further supporting the validity of using pressure measurements to infer different stages of tooth movement.

We performed correlation analysis between force/torque and displacement/rotation angle at days 0, 3, 7, and 15. The results revealed an overall correlation coefficient exceeding 0.99, indicating an exceptionally strong relationship between applied force and tooth displacement (Figure 5h). Figure 5i presents the variation in average force/torque and the corresponding rates of displacement/rotation angle change during the intervals of day 0–3, day 3–7, and day 7–15. Importantly, within the range of forces typically used to induce physiological tooth movement, the displacement of teeth has been shown to exhibit a linear relationship with the applied force. This observation aligns with long‐standing clinical practice and is also supported by previous biomechanical studies.^[^ ^32^, ^33^ ^]^ Therefore, the linear force‐displacement correlation observed in our system reflects the actual biomechanics of orthodontic treatment and confirms that the system operates within clinically relevant force ranges.

## 3 Conclusion

This study proposes a novel, all‐in‐one Orthodontic Force Acquisition System (OFAS) based on a battery‐free iontronic sensing platform, enabling real‐time, in vivo monitoring of 3D orthodontic forces during clear aligner treatment. By incorporating a mechanically robust polyelectrolyte elastomer, an origami‐inspired wireless passive acquisition circuit, and a dual‐layer thermoplastic conformal encapsulation strategy, OFAS achieves high precision (sensing error <1.14%) and long‐term stability under the spatial and mechanical constraints of the oral environment. A 15‐day in vivo trial further demonstrated a strong correlation (R^2^ > 0.99) between the recorded orthodontic force profiles and actual tooth displacement. This work provides a stable and reliable biomechanical feedback mechanism for clear aligner therapy, with the potential to facilitate personalized treatment, reduce complications, and enhance clinical efficacy, marking a significant step toward the digitalization of orthodontic care.

## 4 Experimental Section

### Materials

The following chemicals [EMIM][TFSI] 99%, 1,6‐hexanediol diacrylate (HDDA, H102721), Diphenyl(2,4,6‐trimethylbenzoyl) phosphine oxide (TPO, T107643) were purchased from Aladdin. Ethyleneglycol monomethyl ether acrylate (MEA, E808702) was purchased from Shanghai Macklin Biochemical, PUA (bifunctional aliphatic group) from Chengdu SiChengGuangDian. All chemicals were used as received without further treatment.

### Synthesis of Polyelectrolyte Elastomer

To synthesize PEE, 1.73 g [EMIM][TFSI], 1.04 g MEA, 0.14 g HDDA, and 0.29 g PUA were mixed. Next, A photo‐initiator TPO was added to the mixture of 0.02 g. After being stirred for 1 h at room temperature, the mixture was poured on a commercial abrasive paper (no. 10000#) and cured under 365 nm ultraviolet light(30 W) for 2 h. Finally, the micro‐structured PEE was peeled off the abrasive paper.

### Mechanical and Electrical Characterizations of PEEs

The uniaxial tensile test of PEE was performed utilizing a motorized force tester (ESM 303, Mark‐10), PEE was cut into dumbbell shapes with a gauge length of 15 mm, a gauge width of 3 mm, and a thickness of 1 mm. The loading velocity was fixed at 2 mm min^−1^. For the stability assessment, PEE was fabricated into disk‐shaped specimens with a diameter of 12 mm and a thickness of 1 mm, positioned on filter paper as the substrate, and subjected to a constant pressure of 100 kPa for one week. The mass of the PEE was recorded daily. The Nyquist plots and other electrical characterizations were obtained using circular samples with a thickness of 0.1 mm and a diameter of 5 mm, employing an electrochemical workstation (CHI660E) at an amplitude of 500 mV across a frequency range from 1 Hz to 1 MHz. Prior to testing, the samples were sputter‐coated with copper foil on both sides. The data were analyzed using ZView software in accordance with an equivalent circuit model.

### Characterizations of Iontronic Sensors

The capacitance values of the iontronic sensors were measured using an Inductance‐Capacitance‐Resistance (LCR) digital bridge (TH2829C, Tonghui Inc.) with scanning frequency and voltage of 1 kHz and 1 V, respectively. A motorized force tester (ESM 303, Mark‐10) was used for the pressure‐capacitance response, static compression, and drift tests of the sensor. In drift ratio and drift rate test, a pressure of 500 kPa was applied to the sensor for a duration of 20 000 s. The cyclic compressive test with square waves was programmed such that, during each cycle, the loading stage with a compression of 500 kPa lasted for 40 s, and the unloading stage lasted for 10 s. To assess response/recovery time and output stability under a sine wave load at 1 Hz, the PEE‐based sensor was loaded to cyclic loading and unloading with various frequencies by using A signal generator (AFG1022, Tektronix), The sensor readouts were amplified using operational amplifier circuitry and recorded by a data acquisition card with an exceptionally high sampling rate of 500 s^−1^, as illustrated in Figure S1 (Supporting Information). The precision of the sensor under varying pressure conditions was assessed using a Dead‐Weight Force Standard Machine (DWM‐01, Shaoxing Kente Mechanical & Electrical Co.Ltd). The corresponding capacitance values were measured using an LCR meter (TH2829C, Tonghui Inc.). The calculation method for sensor precision is detailed in Note S1 (Supporting Information). The variation in the initial capacitance of the sensor when attached to surfaces with different curvatures was measured by affixing the sensor to surfaces with curvature radii of 10 mm, 20 mm, 50 mm, 100 mm, and ∞, The results based on serpentine‐trace connection and monolithic structure sensors are compared. The electronic system consists of an MCU (STM32L051K8U6, ST), an NFC chip (NT3H2111, NXP), and a capacitive sensing chip (MPR121QR2, NXP). The NFC chip can receive RF signal energy while simultaneously transmitting signals. An external storage capacitor connected to the NFC chip helps stabilize the circuit's power supply voltage.

### Integration and Encapsulation of the OFAS

The 3D‐printed dental arch model representing the target displacement state was placed into a vacuum thermoforming machine (Jinguang, China) along with a 0.1 mm‐thick TPU film (Shore hardness 85A, Xinwang Thermoplastic Materials, China) for vacuum forming (Figure S12, Supporting Information). After the initial forming, the sensing circuit was positioned on the target tooth. Subsequently, a 0.6 mm‐thick orthodontic resin sheet (Rigid film, Juli Medical Devices Co., Ltd., China) was placed over the model for a second vacuum thermoforming step. Following the subsequent forming, the excess material outside the crown‐adhering area was trimmed away, and the edge regions were polished using a rotary grinder. The overall effective thickness of the orthodontic resin film, encapsulation layer, and sensor was ≈0.9 mm. For comparison, conventional metal brackets were typically 2–3 mm in thickness, while commercial clear aligners generally ranged from 0.5 to 1.5 mm in thickness. Therefore, the design falls within the reasonable thickness range of the commercial clear aligners and can effectively ensure patient comfort during wear.^[^ ^34^ ^]^ Benefiting from the sensing material's high Young's modulus and fracture strain, as well as the use of serpentine interconnects between sensing units, the sensor exhibits excellent conformability to curved surfaces (as shown in Figure 2c,h), allowing it to adhere well to the high‐modulus membrane without compromising sensing functionality. Although the encapsulation introduces a small initial pressure on the sensor, this effect can be corrected using the sensor's calibration curve and becomes negligible under high pressure conditions (Figure S15, Supporting Information).

### Electronic System Design and Characterization

The NFC antenna was designed with dimensions of 7 mm × 7 mm (length × width), consisting of 6 turns. The track width and the gap between adjacent tracks were both 300 µm. The coil pattern was alternately distributed on the top and bottom layers of the substrate. In these experiments, two methods were used for wireless power supply: a card reader (CU100‐NTAG, Guangzhou Shengju Intelligent Technology) and a smartphone (K40S, Redmi). At a testing distance of 5 mm between the antenna and the power source, the OFAS (On‐Field Antenna System) consistently maintained a stable power supply voltage of 3 to 3.1 V, ensuring reliable operation for data processing, wireless communication, and sensing modules. For antenna performance testing under various deformation conditions, a miniVNA was used. An external reading antenna was positioned 5mm from the antenna of the circuit under test. The resonance frequency shifts of the circuit antenna were recorded and saved within the 5–25MHz range. In the comparative experiments on signal transmission stability between NFC‐based and LC‐based systems, data from the OFAS and LC antennas were collected using an NFC card reader and a miniVNA, respectively, under unloaded sensor conditions. The experiments monitored the variation in sensing signals as the relative position between the center points of the reading antenna and the sensing antenna was offset by 0, 2, 4, and 6 mm. This experiment was conducted to evaluate the impact of spatial misalignment between the reader and receiving antennas on signal transmission stability. Specifically, it aims to demonstrate that LC‐based systems exhibit noticeable shifts in resonant frequency under coil displacement, whereas NFC‐based systems maintain stable performance, highlighting their robustness to positional variations in practical applications.

### Cytotoxicity Characterization Assay

For the biocompatibility assessment, human gingival fibroblasts (HGFs) were cultured in Dulbecco's Modified Eagle Medium (DMEM) supplemented with 15% fetal bovine serum (FBS), 100 U mL^−1^ penicillin, and 100 µg mL^−1^ streptomycin. Cells were seeded onto three types of samples: 10 mm PEE membranes, clear aligners, and clear aligners integrated with the OFAS circuitry. After incubation for 1, 3, 7, and 14 days, cell viability was evaluated using the Cell Counting Kit‐8 (CCK‐8) assay. Absorbance at 450 nm was measured using a microplate reader to quantify HGF viability in each well. Additionally, HGFs were seeded into 24‐well plates and cultured for 24 h. The cells were then stained with Calcein AM/PI working solution and incubated at 37 °C in the dark for 30 min. Fluorescence imaging was subsequently performed using a fluorescence microscope to assess live/dead cell staining outcomes.

### In Vitro Simulation Experiments

In vitro dentognathic deformity was achieved by Typodont simulation jaw frame. Three deformity conditions of rotation, translation, and tilt were simulated by the plasticity of the paraffin around a single tooth (Figure S13, Supporting Information). The wires used for heating were pre‐buried around the root of the target tooth to simulate alveolar bone reconstruction during orthodontic treatment. After wearing orthodontic sensor braces, the heating program was started by a DC Power Supply (2231A‐30‐3, Keithley Instruments, Cleveland, OH, USA), and the sensing data during tooth movement were recorded by the acquisition circuit as shown in Figure S13 (Supporting Information).

### In Vivo Experiment

The human dental study was approved by the Biomedical Ethics Committee of Peking University School and Hospital of Stomatology (Approval No. 2025107006). All participants were informed of the testing procedures and potential risks, and written informed consent was obtained prior to participation. No signs of oral ulcers or related conditions were observed. A single orthodontic cycle was selected for OFAS wear testing. During this cycle, the prescribed tooth movement involved an inward translation of 200 µm accompanied by a 1° rotation along the tooth's central axis. Data acquisition was performed using an NFC reader. Participants wearing the clear plastic aligners were instructed to remove the appliance during meals and oral hygiene activities and to clean the aligner during these periods. To minimize potential interference from circadian rhythms and inconsistencies in measurement timing, force data were consistently collected once daily within a fixed time window (12:00–14:00). Given the subtle nature of orthodontic tooth movement, a single measurement per day was deemed sufficient. The selected time window also facilitated the coordination of intraoral scanning procedures in a clinical setting. Spatial data of the maxillary dentition were acquired on days 0, 3, 7, and 15, and temporal changes in tooth position were analyzed through geometric comparisons.

## Conflict of Interest

The authors declare no conflict of interest.

## Author Contributions

J.G., Q.N., and J.X. contributed equally to this work. J.G., S.C., T.P., and Y.C. conceived the ideas. J.G., R.W., Q.N., and Y.C. wrote the manuscript. J.G., J.X., R.W., and Q.N. designed the experiment. J.G., J.X., Q.N., X.W., L.W., J.G., and H.Z. performed experiments and analyzed the experiments data. All authors reviewed and provided constructive feedback on the manuscript.

## Supporting information



Supporting Information

## Data Availability

The data that support the findings of this study are available from the corresponding author upon reasonable request.
