# Supplementary material for: All‐In‐One Iontronic Sensing Aligner for High‐Precision 3D Orthodontic Force Monitoring
Source: Adv Sci (Weinh). 2025 Sep 8;12(43):e11984. doi: 10.1002/advs.202511984 (PMC12631939; doi:10.1002/advs.202511984)
Supplement: Supplementary file 1 — Supporting Information [file ADVS-12-e11984-s001.docx]

Supporting Information

All-in-One Iontronic Sensing Aligner for High-precision 3D Orthodontic Force Monitoring

Jiahao Guo, Qin Niu, Jilei Xu, Liyan Wang, Xiangyang Wang, Jiangdong Gong, Hanfei Zhu, Ruojiang Wang*, Yu Chang*, Si Chen*, Tingrui Pan*

**The file includes:**

Supplementary Note 1

Supplementary Tables 1

Supplementary Figures 1 to 14

**Supplementary Note 1. Method for Measuring and Calculating Sensor Precision.**

Precision indicates how closely repeated measurements match each other^1,2^. The sensor under test was placed in a deadweight-type force standard machine, where three cycles of stepwise loading were applied. The corresponding capacitance signals were recorded using an LCR meter.

The precision at a specified pressure $P_{i}$ was calculated using the following formula^2^:

$$Precision_{P_{i}}=\frac{\sigma C_{n}}{\bar{C_{n}}}$$

where $C_{n}$ represents the capacitance values measured under pressure $P_{i}$​, $\sigma C_{n}$ is the standard deviation, and $\bar{C_{n}}$​​ is the mean of the capacitance values at that pressure.

| Method | Wireless? | accuracy | Multi-axis | Dynamic? | Totally wearable? | Full scale |
| --- | --- | --- | --- | --- | --- | --- |
| LC sensing^3^ | YES | ±0.2N  (8%) | 1-axis | YES | YES | 2.5N  (278kPa) |
| LC sensing^4^ | YES | 1% | 1-axis | YES | YES | 650kPa |
| High density sensors array ^5^ | NO | \ | 5-axis | YES | NO | 250kPa |
| Pressure-Sensitive Film ^6^ | YES | ±0.1N | 1-axis | NO | NO | 2N |
| Stress measuring chip^7^ | NO | 10% | 3-axis | YES | NO | 0.1-1N  (250kPa) |
| single force sensor^8^ | NO | ±0.05N  (1%) | 1-axis | YES | NO | 5N  (50kPa) |
| Multi-axis 3D mechanical measurement system^9^ | NO | 1.66% | 6-axis | YES | NO | \ |
| This work | YES | 0.4% | 3-axis | YES | YES | 4N  (1000kPa) |

**Supplementary Table 1. Technical comparison of Orthodontic force measurement devices.**


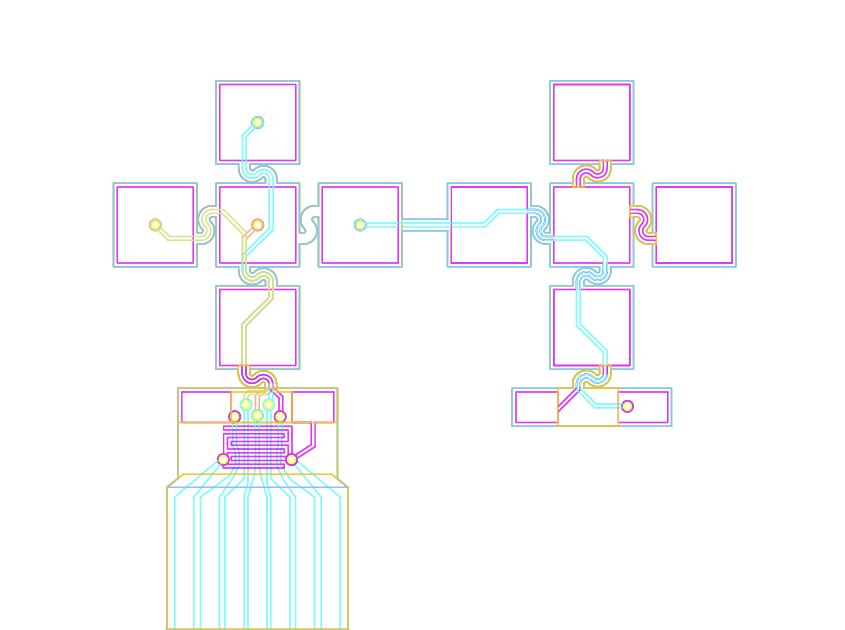


**Figure S1.** Schematic diagram of a FPC electrode array design based on cross structures.

**
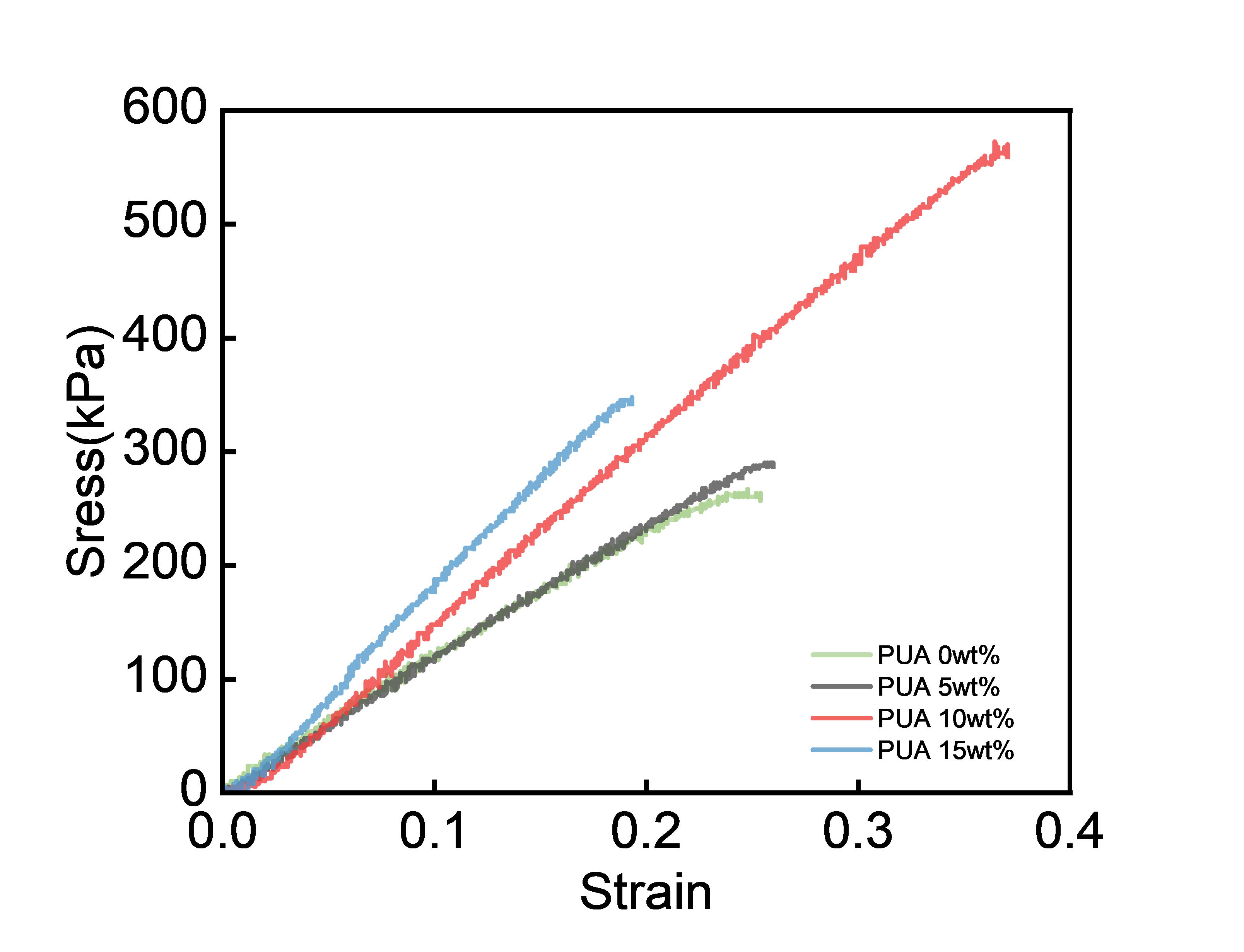
**

**Figure S2.** Tensile stress–strain results of the PEE with various PUA concentrations.


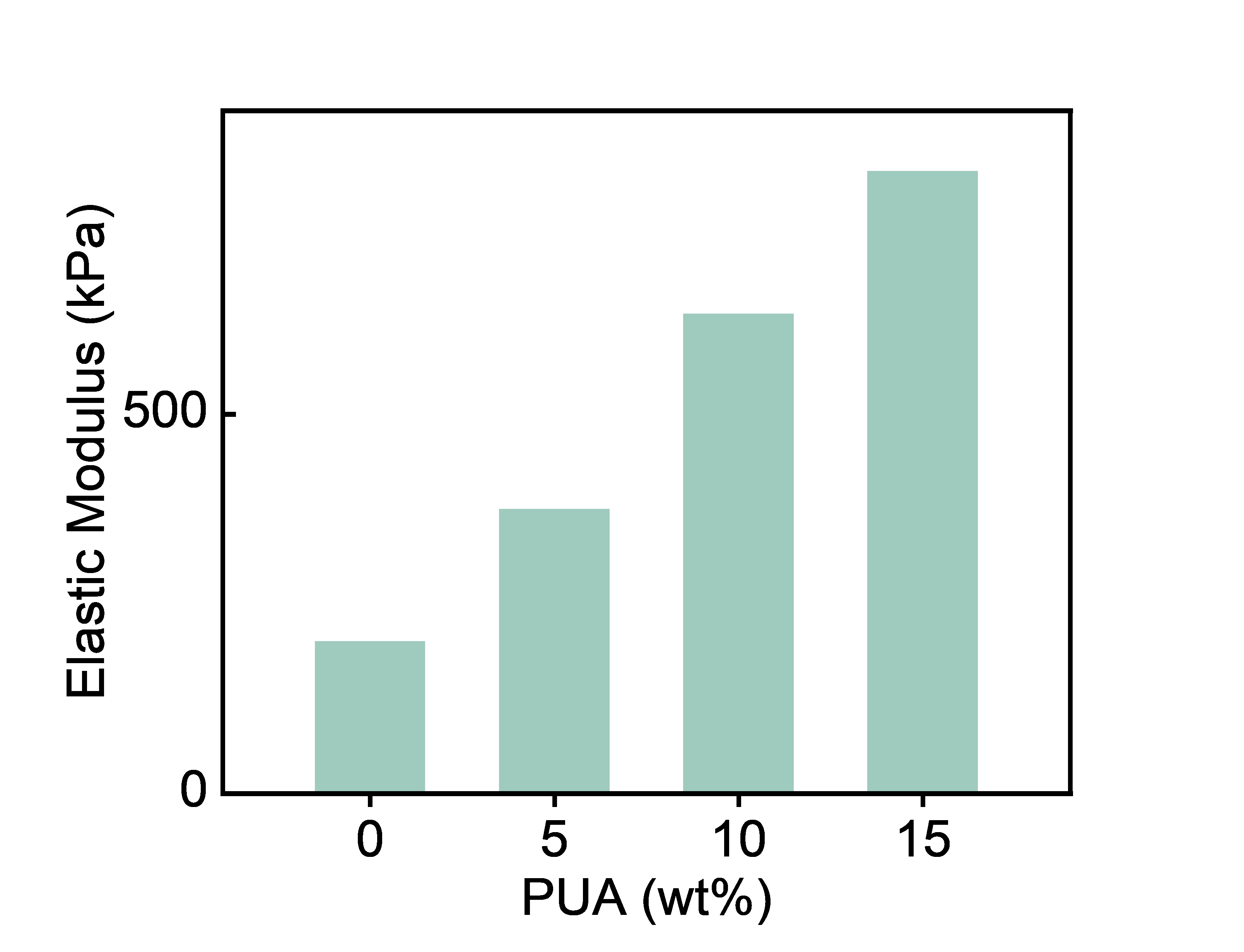


**Figure S3.** The variations of elastic modulus with crosslink density.


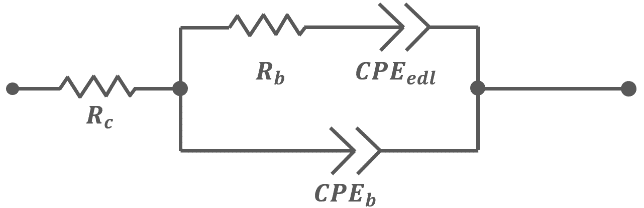

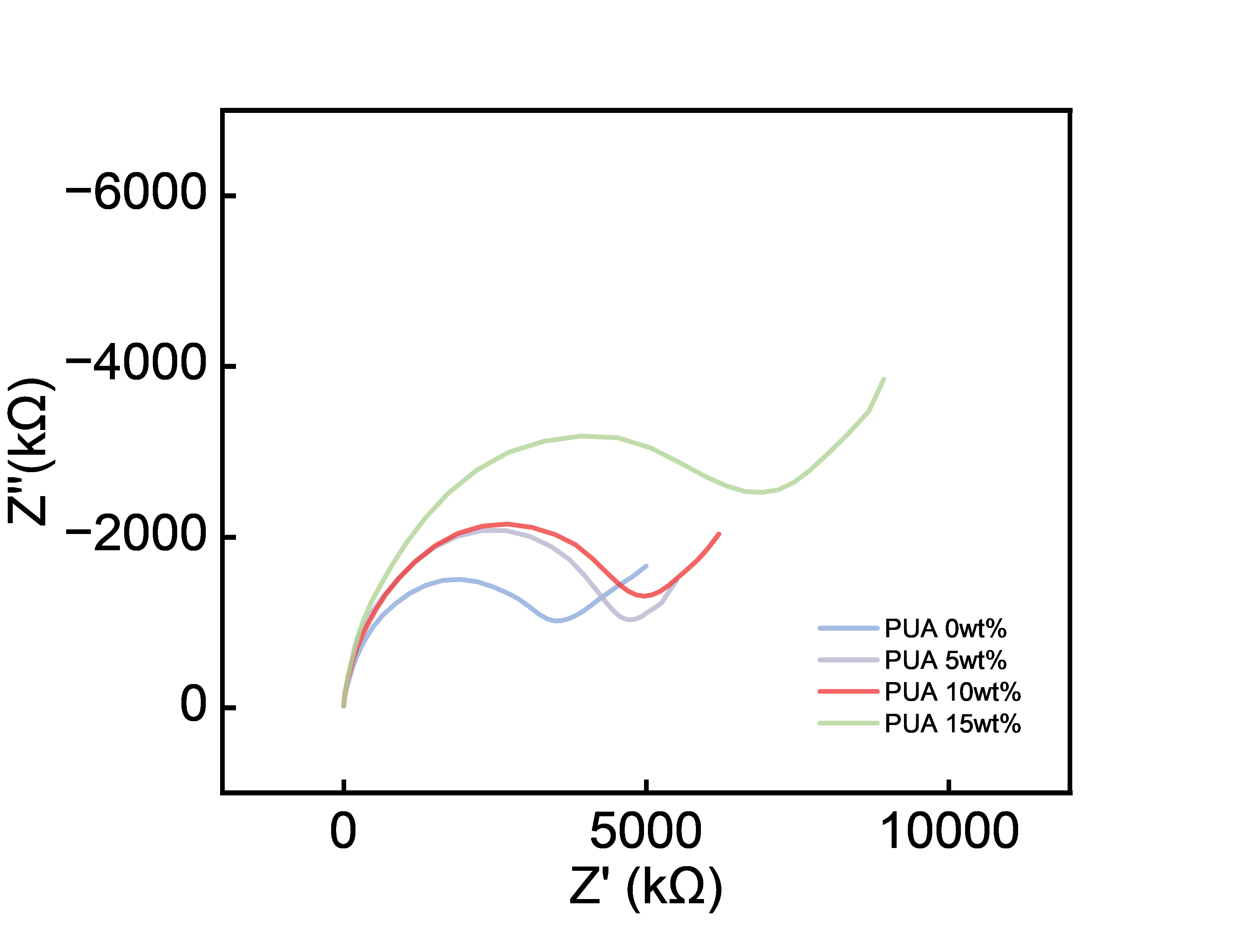


**Figure S4.** Nyquist plots of PEEs with different crosslink densities. The inset shows the circuit model used for fitting the data.


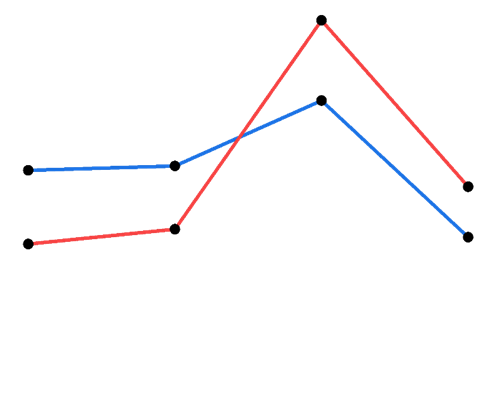

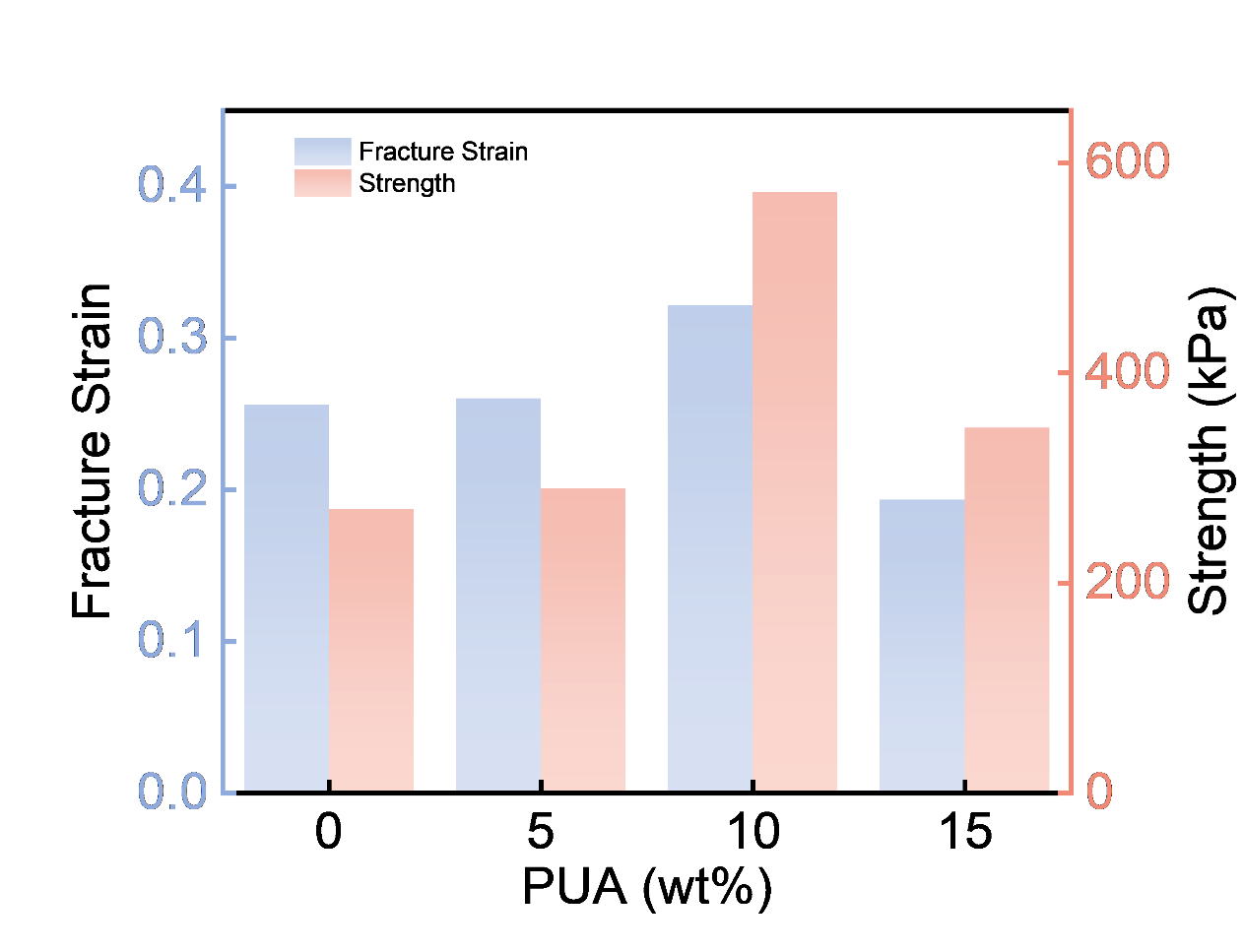


**Figure S5.** The variations of fracture strain and strength with crosslink density.


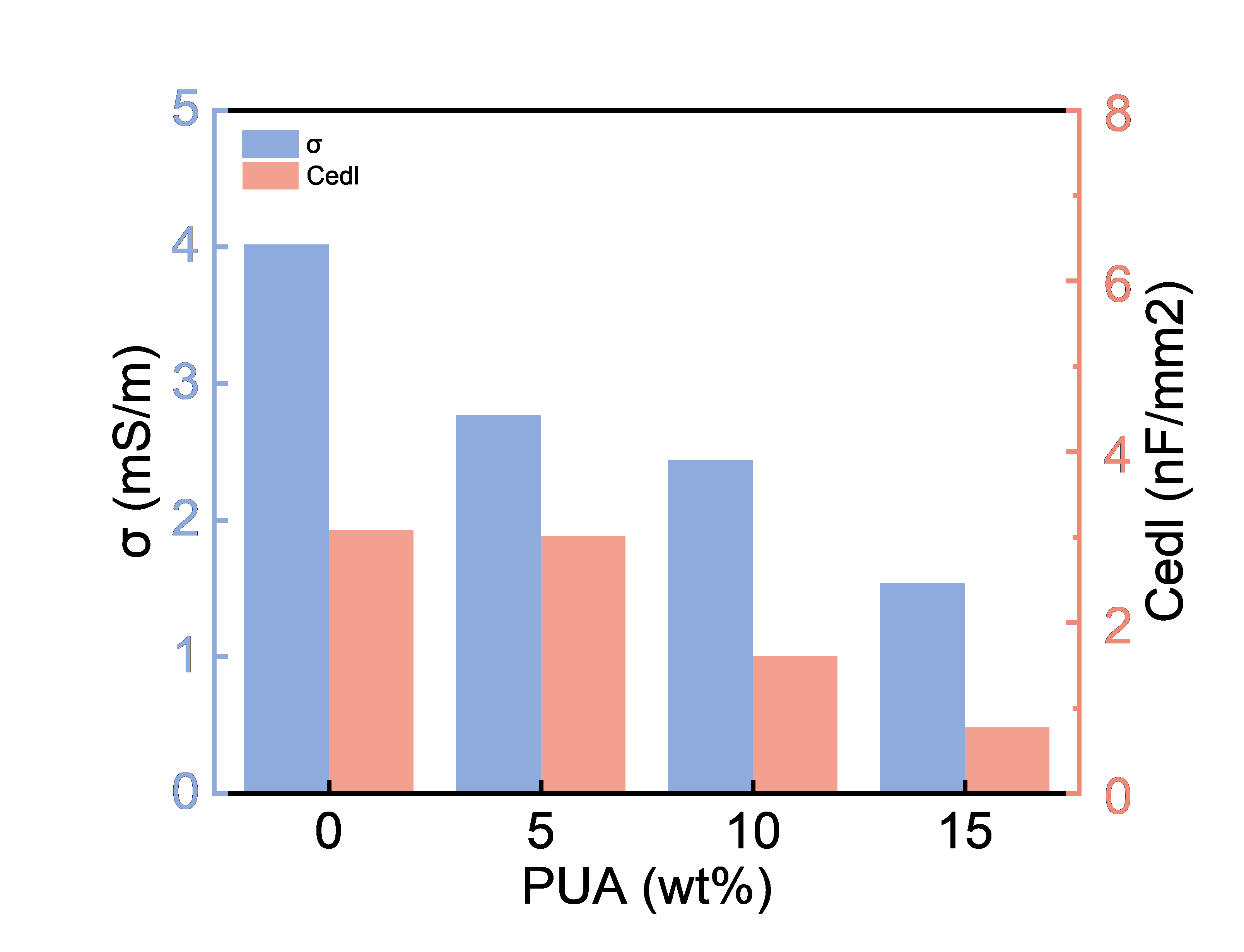


**Figure S6.** The variations of ionic conductivity and the capacitance of EDL per unit area with crosslink density.


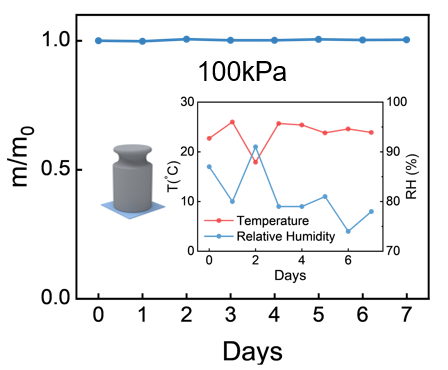


**Figure S7.** Time-dependent mass variation of the PEE under continuous pressure of 100 kPa. Inset: visual comparison of the PEE and the underlying filter paper before and after the test. Scale bar: 1 cm..


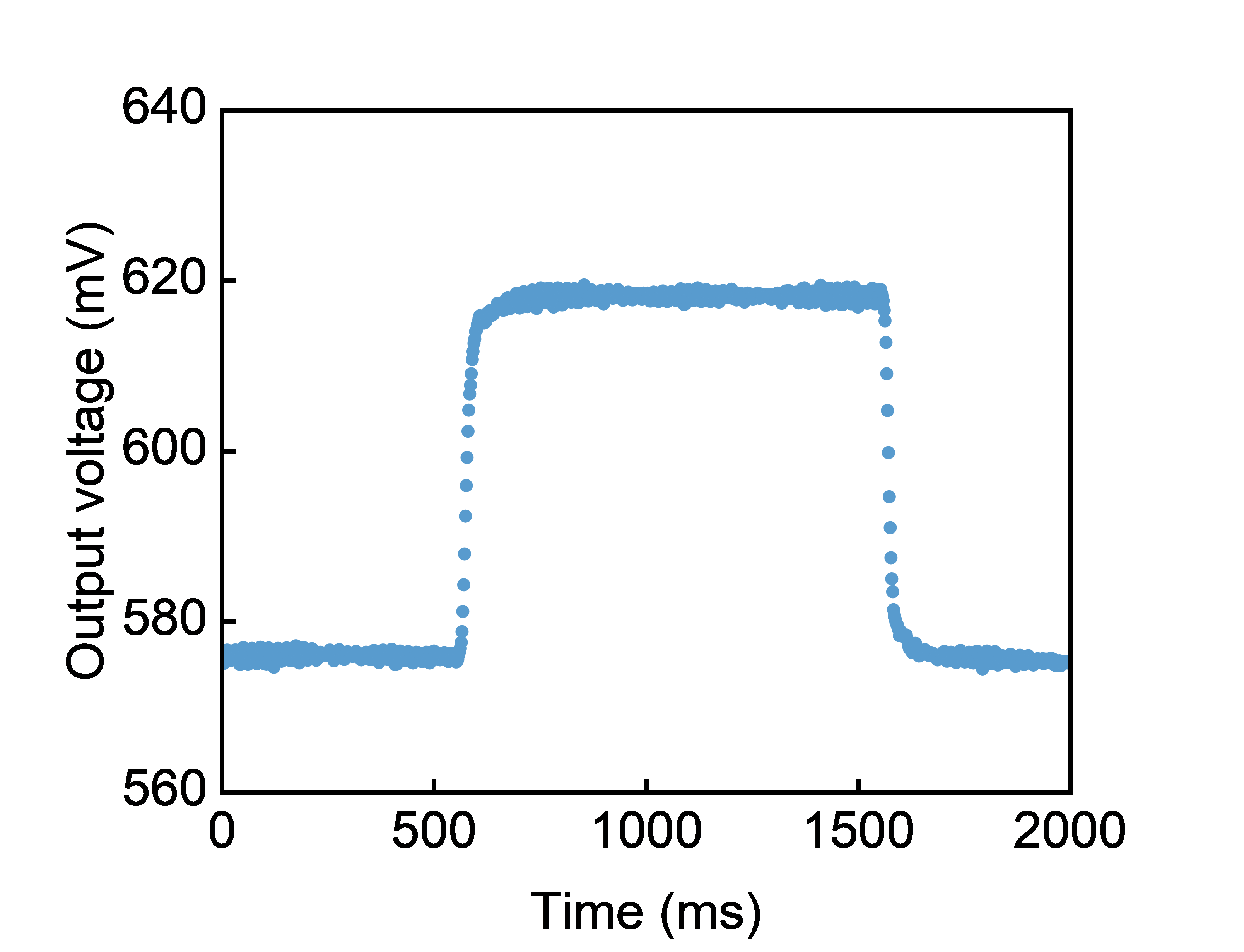


**Figure S8.** The response and reset times of the CSA.


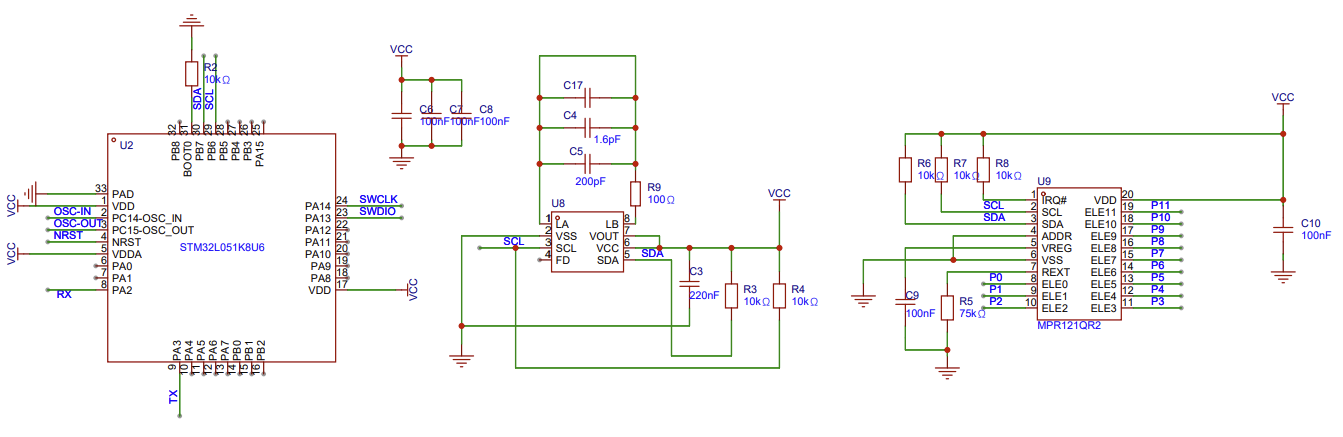


**Figure S9.** Schematic diagram of the OFAS system circuit.


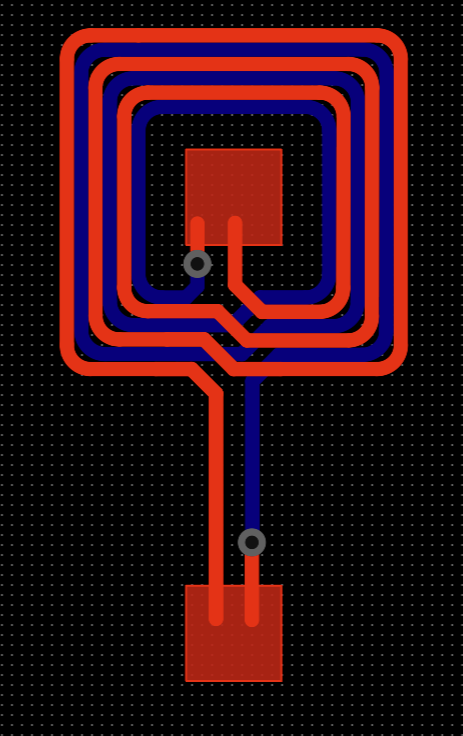
**
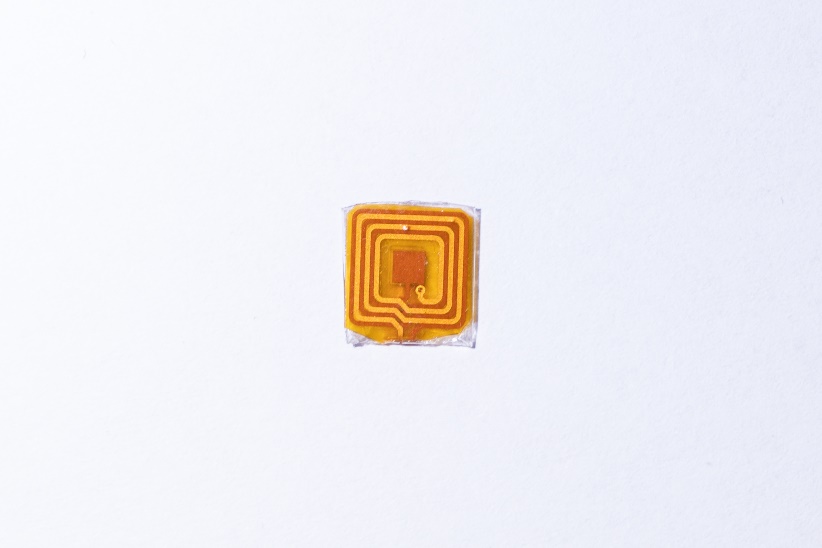
**

**Figure S10.** Circuit diagram and physical image of the LC sensor.

**
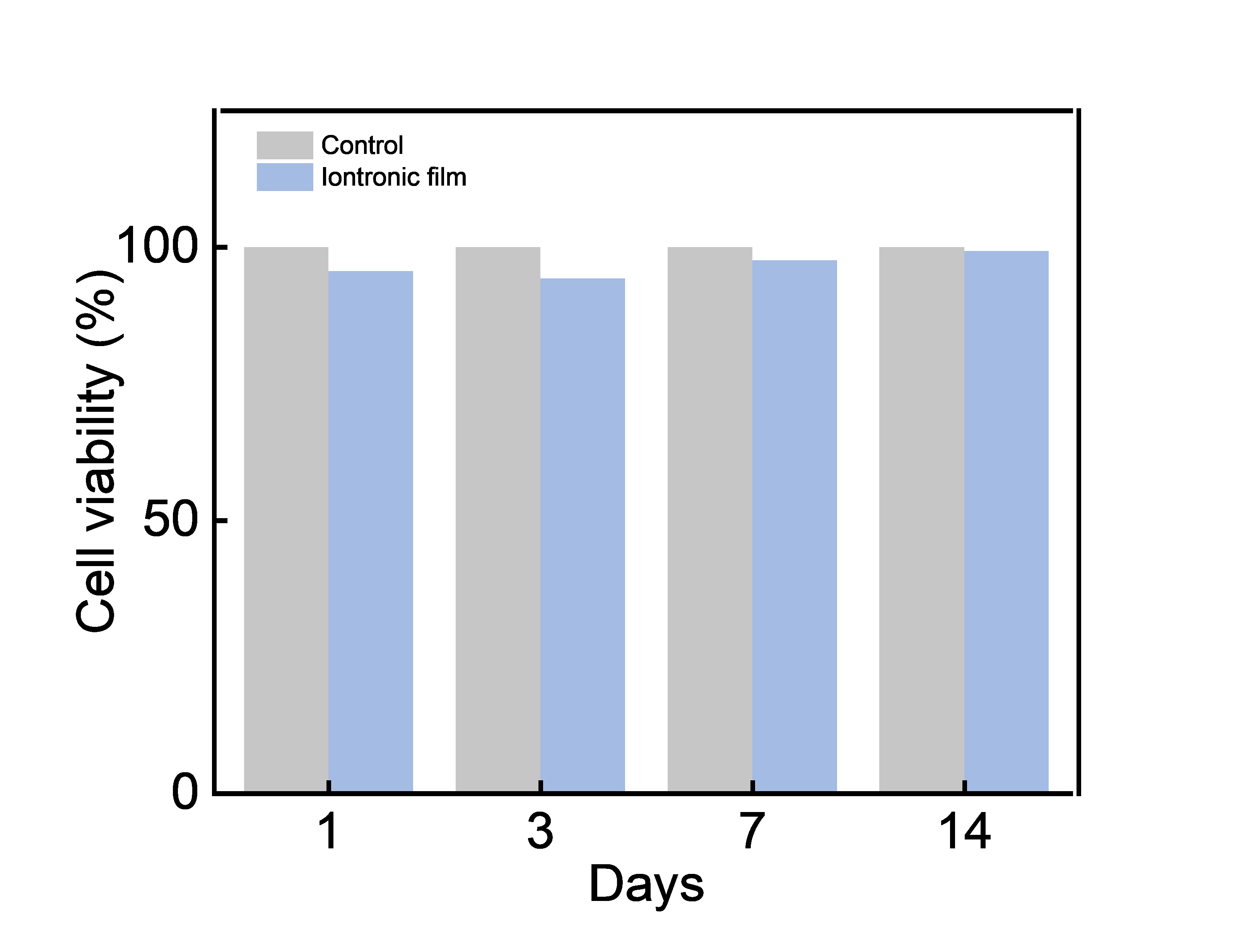
**

**Figure S11.** Cell viability assessment of Iontronic film.


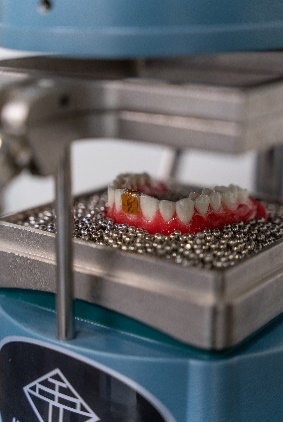

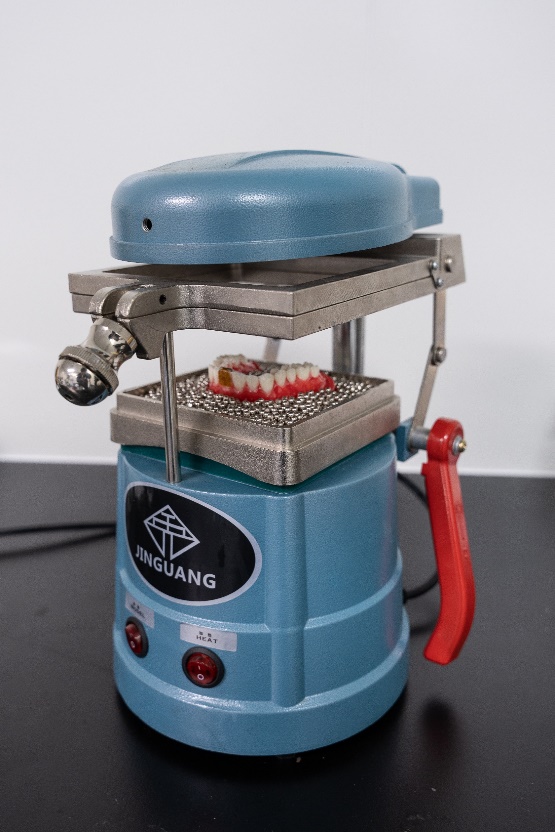


**Figure S12.** Vacuum thermoforming apparatus .


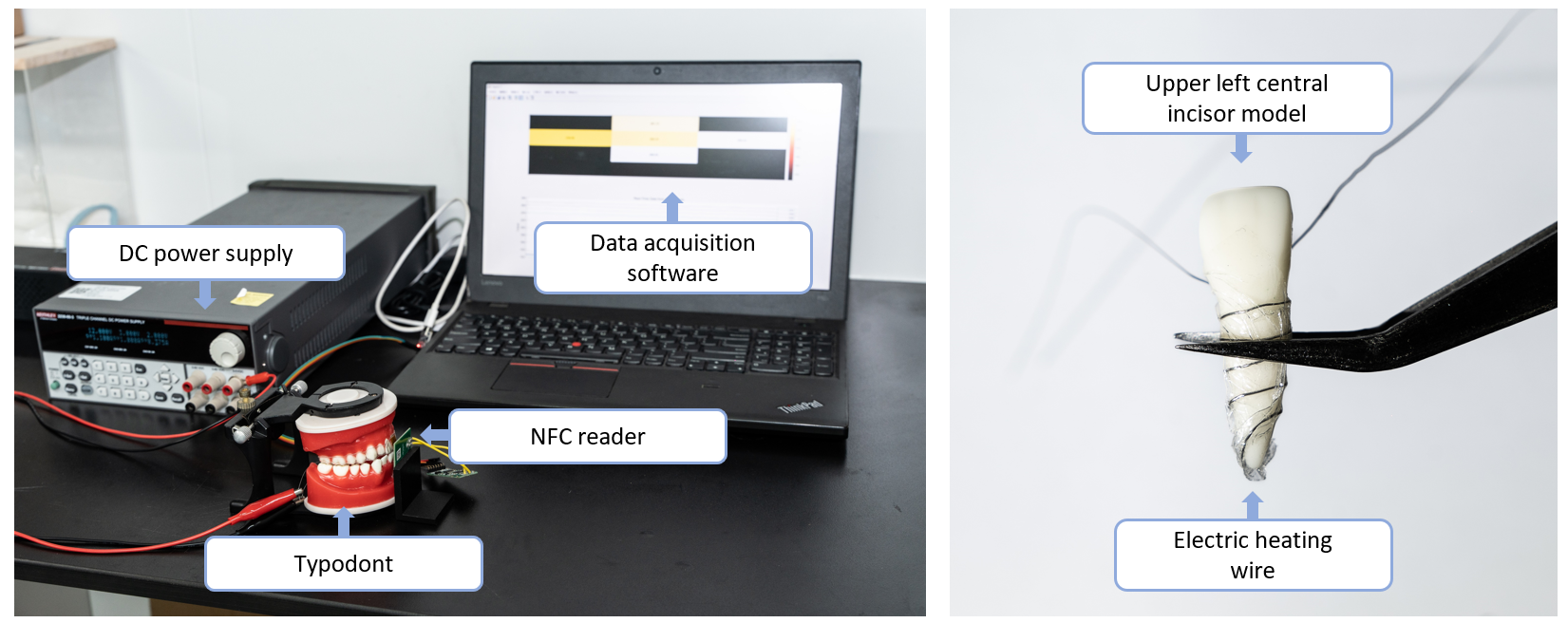


**Figure S13.** Schematic of the simulated maxillary testing setup (left); schematic of the tooth model wrapped with a heating wire (right), which is used to locally heat the wax surrounding the root of the ta3rget tooth to accelerate its displacement.


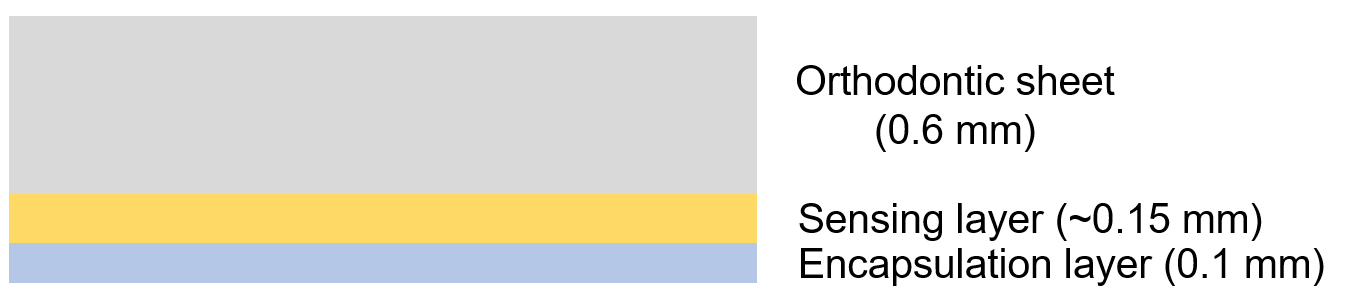
 **
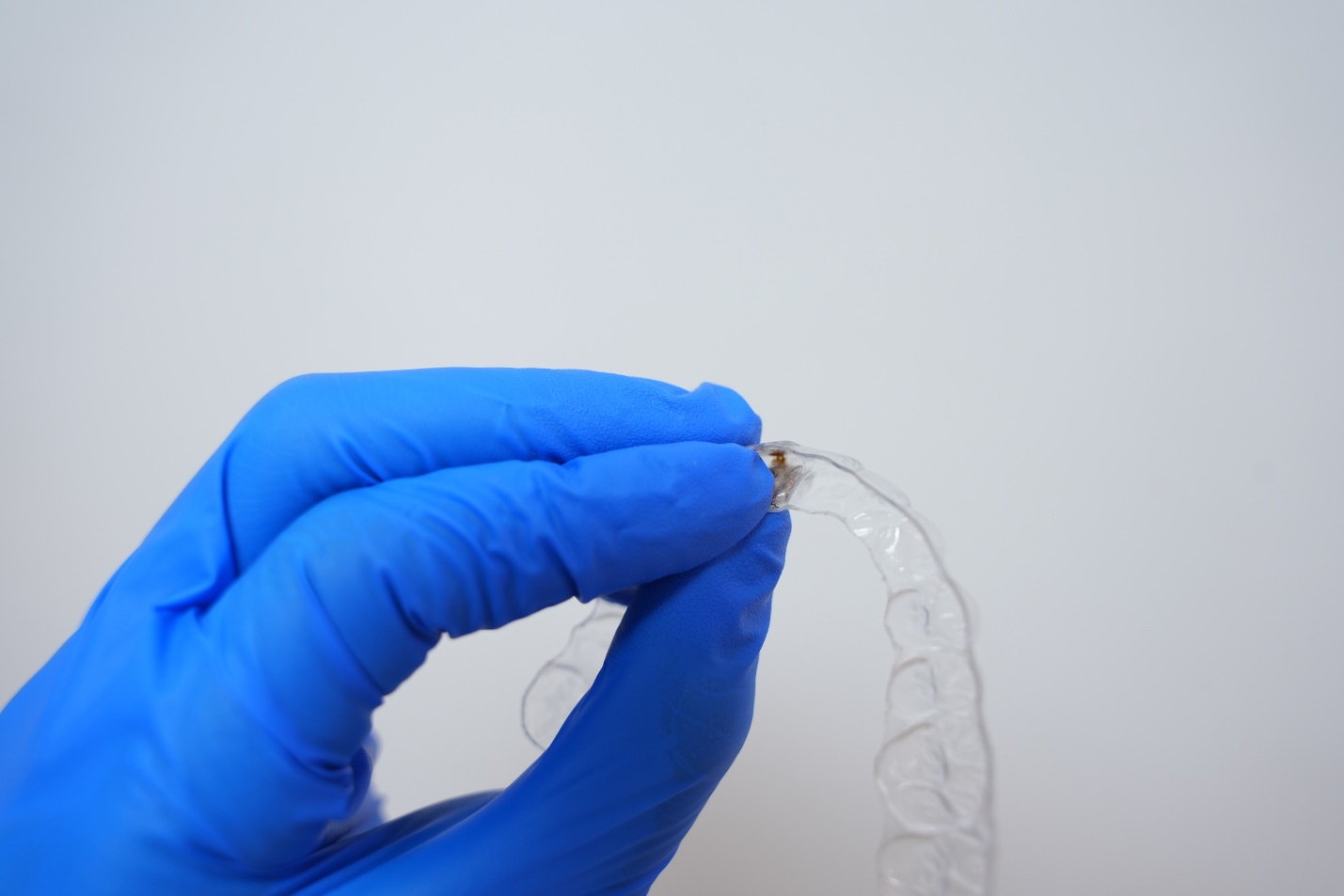
**

**Figure S14.** Schematic illustration of OFAS encapsulation (left); side view of the actual OFAS device (right).

**
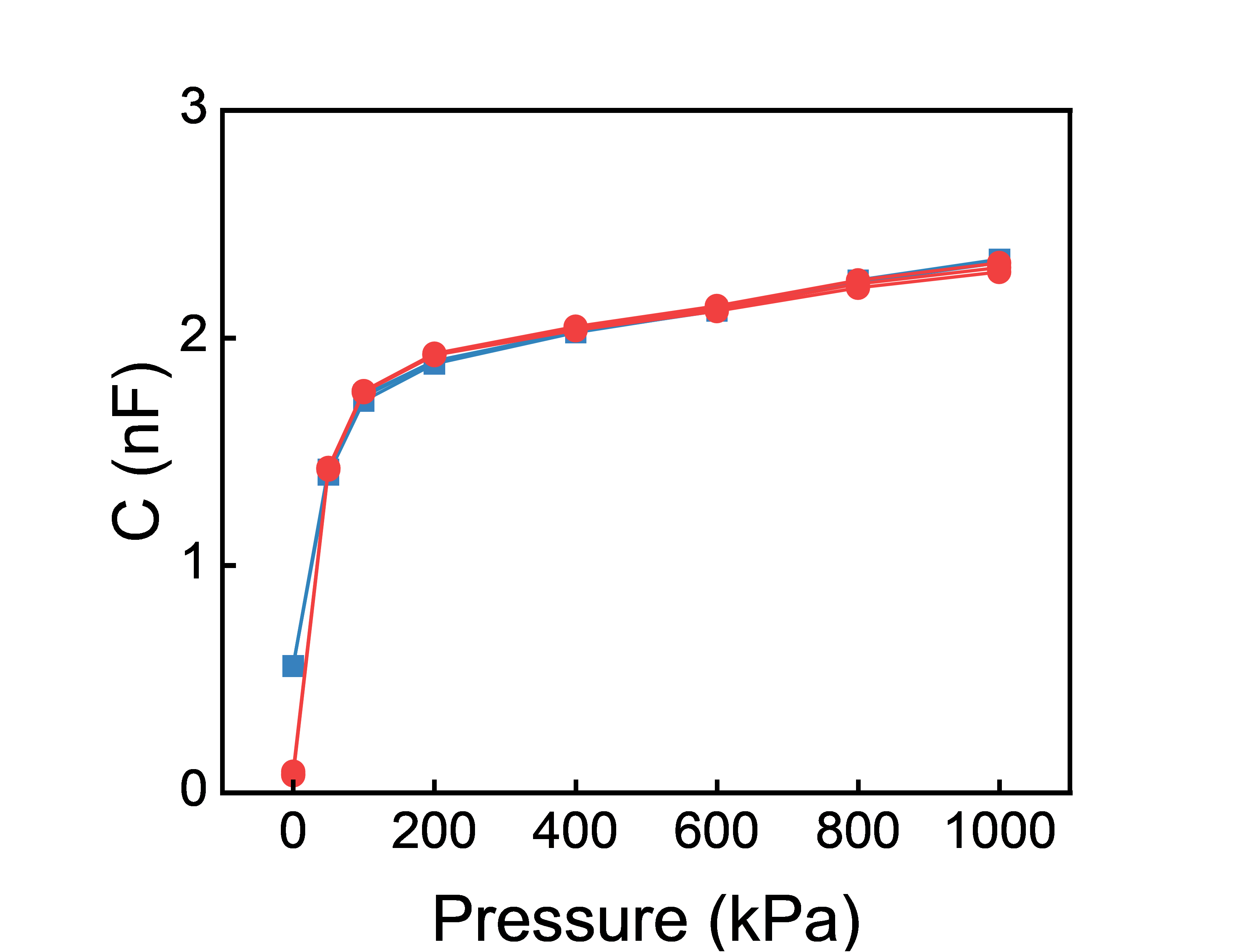
**

**Figure S15.** Capacitance–pressure response curves of the CISA before (red) and after (blue) encapsulation. Aside from a shift in the initial capacitance value, no significant differences were observed between the two curves under applied pressures (n = 3).

**Supplementary References**

1. Shi, L., Li, Z., Chen, M., Zhu, T., and Wu, L. (2023). Ultrasensitive and Ultraprecise Pressure Sensors for Soft Systems. Advanced Materials *35*. 10.1002/adma.202210091.

2. China, S.A.o. (2015). Test methods of the performances for pressure transducer/sensor.

3. Wang, S., Xu, B., Wang, X., Li, X., Pu, B., Chen, G., Han, J., Xiao, S., Fan, Y., and Li, J. (2024). Wireless measurement of orthodontic forces in invisible aligners. Medicine in Novel Technology and Devices *21*. 10.1016/j.medntd.2023.100282.

4. Song, J., Yang, R., Shi, J., Chen, X., Xie, S., Liao, Z., Zou, R., Feng, Y., Ye, T.T., and Guo, C.F. (2025). Polyelectrolyte-based wireless and drift-free iontronic sensors for orthodontic sensing. Science Advances *11*, eadu6086.

5. Sun, C., Li, S., Zhou, Z., Chen, X., Liu, Z., Liao, S., Xing, J., Sun, H., Cheng, Y., Yi, Z., et al. (2024). Conformal Iontronic Sensing Clear Aligner. Advanced Functional Materials *34*. 10.1002/adfm.202408376.

6. Zamani, N.S.M., Ashari, A., Ali, S.H.M., Gan, K.B., How, R.A.W.M., Wahab, R.M.A., Mohamed, A.M.F.S., Sinnasamy, S., and Mokhtar, M.H.H. (2022). Distributed Force Measurement and Mapping Using Pressure-Sensitive Film and Image Processing for Active and Passive Aligners on Orthodontic Attachments. Ieee Access *10*, 52853-52865. 10.1109/access.2022.3175210.

7. Shi, Y., Ren, C., Hao, W., Zhang, M., Bai, Y., and Wang, Z. (2012). An Ultra-Thin Piezoresistive Stress Sensor for Measurement of Tooth Orthodontic Force in Invisible Aligners. Ieee Sensors Journal *12*, 1090-1097. 10.1109/jsen.2011.2166065.

8. Liu, L., He, B., Zhuang, J., Zhang, L., and Lv, A. (2017). Force measurement system for invisalign based on thin film single force sensor. Measurement *97*, 1-7. 10.1016/j.measurement.2016.11.018.

9. Kaur, H., Truong, J., Heo, G., Mah, J.K., Major, P.W., and Romanyk, D.L. (2021). An in vitro evaluation of orthodontic aligner biomechanics around the maxillary arch. American Journal of Orthodontics and Dentofacial Orthopedics *160*, 401-409. 10.1016/j.ajodo.2021.04.005.
